# Unprocessed U1 snRNAs as a biomarker of INTS11- and BRAT1-related neurodevelopmental disorders

**DOI:** 10.1186/s13073-026-01667-1

**Published:** 2026-05-12

**Authors:** Beatrice Valtorta, Zuzana Polackova, Reza Maroofian, Aveeva Herold, Irem Karagoz, Maha S Zaki, Denisa Bronisova, Meijiang Liao, Mina Zamani, Annarita Scardamaglia, Nine Collomb, Lidia Lopez-Jimenez, Maria J Barrero, Julian Schröter, Steffen Syrbe, Marion Heidi Vallanger, Sofia Douzgou Houge, Yasemin Alanay, Ozlem Akgun-Dogan, Julie Vogt, Michael Muriello, Yvonne M C Hendriks, Alexandra Afenjar, Nadirah Damseh, Rauan Kaiyrzhanov, Marcello Niceta, Marco Tartaglia, Manju A Kurian, Nataliya Di Donato, Grace Yoon, Henry Houlden, Éric Samarut, Hana Hanzlikova

**Affiliations:** 1 Laboratory of Genome Dynamics, Institute of Molecular Genetics of the Czech Academy of Sciences, Videnska 1083, Prague 4, 142 20 Czech Republic; 2 Faculty of Science, Charles University in Prague, Prague 2, Czech Republic; 3 Department of Neuromuscular Diseases, Queen Square Institute of Neurology, University College London, London, UK; 4 Department of Neuroscience, Faculty of Medicine, University of Montreal, Montreal, QC Canada; 5 University of Montreal Hospital Research Center (CRCHUM), Montreal, QC Canada; 6 Department of Clinical Genetics, Human Genetics and Genome Research Institute, National Research Centre, Cairo, Egypt; 7 Instituto de Investigación de Enfermedades Raras, Instituto de Salud Carlos III, Madrid, Spain; 8 Undiagnosed diseases program SpainUDP, Madrid, Spain; 9 Center for Child and Adolescent Medicine, Division of Pediatric Epileptology, Heidelberg University, Heidelberg, Germany; 10 Department of Medical Genetics, Haukeland University Hospital, Bergen, Norway; 11 Acibadem Mehmet Ali Aydinlar University Rare Diseases and Orphan Drugs Application and Research Center (ACURARE), Istanbul, Turkey; 12 Division of Pediatric Genetics, Department of Pediatrics, School of Medicine, Acibadem Mehmet Ali Aydinlar University, Istanbul, Turkey; 13 West Midlands Regional Genetics Service, Birmingham Women’s and Children’s NHS Foundation Trust, Birmingham, UK; 14 Division of Genetics, Department of Pediatrics, Medical College of Wisconsin, Milwaukee, WI USA; 15 Department of Clinical Genetics, Leiden University Medical Center, Leiden, The Netherlands; 16 Clinical Genetics Unit, Reference Center for Cerebellar Malformations and Congenital Diseases, APHP, Armand-Trousseau Hospital, Sorbonne University, Paris, France; 17 Department of Pediatrics and Genetics, Al Makassed Hospital and Al-Quds University, Jerusalem, Palestine; 18 Molecular Genetics and Functional Genomics, Ospedale Pediatrico Bambino Gesù, IRCCS, Rome, Italy; 19 Developmental Neurosciences, Zayed Centre for Research into Rare Disease in Children, UCL Great Ormond Institute of Child Health, London, UK; 20 Institute for Clinical Genetics, University Hospital Carl Gustav Carus at the Technische Universität Dresden, Dresden, Germany; 21 Department of Pediatrics, Division of Clinical and Metabolic Genetics, The Hospital for Sick Children, University of Toronto, Toronto, Canada; 22 Institute of Animal Pathology, Vetsuisse Faculty, University of Bern, Bern, Switzerland

**Keywords:** INTS11, BRAT1, U snRNAs, Neurological disease

## Abstract

**Background:**

Disrupted RNA processing is increasingly recognized as a key driver of severe neurodevelopmental disorders. Variants in the Integrator catalytic subunit INTS11 and its binding partner BRAT1 lead to clinically overlapping phenotypes, yet only the molecular function of INTS11 has been relatively well characterized. In contrast, the mechanistic contribution of BRAT1 to RNA metabolism and disease has remained unclear, leaving major gaps in variant interpretation and diagnostic classification.

**Methods:**

We employed an integrated genetic, molecular, and in vivo approach to investigate the impact of *INTS11* and *BRAT1* mutations on U small nuclear RNA (U snRNA) processing. Patient-derived fibroblasts and lymphoblastoid cells were analysed by western blotting, RT-qPCR and fluorescence in situ hybridization to assess U1 snRNA 3′-end processing and nuclear retention. To validate the functional consequences of Integrator deficiency in vivo, we generated and characterized an *ints11* knockout zebrafish model.

**Results:**

We identified novel biallelic variants in *INTS11* and *BRAT1* in individuals with overlapping neurodevelopmental features. While defective snRNA processing is anticipated in INTS11 deficiency, this study provides the first direct demonstration of impaired U1 snRNA processing across multiple *INTS11*-mutated patient cells. Critically, we show that *BRAT1* mutations also compromise U1 snRNA 3′-end processing, leading to nuclear accumulation of unprocessed transcripts. These findings provide direct evidence of BRAT1’s role in RNA processing and establish Integrator dysfunction as a primary pathogenic mechanism in BRAT1-associated neurological disease. The magnitude of U1 snRNA misprocessing closely correlates with clinical severity across the BRAT1 cohort, highlighting its potential as a diagnostic biomarker. Consistently, the *ints11* knockout zebrafish model recapitulates core patient features – including microcephaly, neurodevelopmental defects, and U snRNA processing defects – further validating the causal role of Integrator deficiency in vivo.

**Conclusions:**

Our results redefine BRAT1-associated neurological disorders as Integrator-related diseases driven by RNA processing defects. Nuclear accumulation of unprocessed U1 snRNAs emerges as a robust biomarker for variant interpretation, disease severity, and patient stratification, particularly in BRAT1 cases. These findings broaden the clinical and molecular spectrum of Integrator dysfunction and provide a foundation for improved diagnostic and translational approaches.

**Supplementary Information:**

The online version contains supplementary material available at 10.1186/s13073-026-01667-1.

## Background

The Integrator complex subunit 11 (INTS11) encodes the endonuclease catalytic subunit of the RNA polymerase II (RNAPII)-associated Integrator complex, a multifunctional regulator of RNA processing and transcription [1]. Originally described as a 12-subunit, metazoan-specific complex responsible for 3′-end processing of U small nuclear RNAs (U snRNAs), the Integrator complex was later expanded to include at least 15 subunits (INTS1–15) [2–5]. The catalytic core, or cleavage module, comprises the endonuclease INTS11, the pseudoenzyme INTS9, and the scaffold protein INTS4 [6–8]. Beyond U snRNA processing, INTS11 has been implicated in the cleavage of diverse RNA species, including enhancer RNAs (eRNAs), long-noncoding RNAs (lncRNAs), PIWI-interacting RNAs (piRNAs), and telomerase-associated RNA [2, 9–14]. Additionally, the Integrator complex has emerged as a key regulator of transcription, particularly through its control of promoter-proximal pausing of RNAPII, a function critical for embryogenesis and cell differentiation [1, 4].

Mutations in Integrator subunits are increasingly recognized as causes of human genetic disorders [15]. Pathogenic variants in *INTS1* and *INTS8* are associated with autosomal-recessive neurodevelopmental syndromes characterized by developmental delay, cognitive and motor impairments, and dysmorphic features, while *INTS13* mutations have been linked to autosomal-recessive ciliopathies and *INTS15* mutations to autosomal-dominant eye diseases with variable manifestations [16–19]. More recently, biallelic *INTS11* mutations have been identified in patients presenting with intellectual disability (ID), microcephaly, motor impairment and cerebellar atrophy [20–22], while *INTS6* mutations have been linked to language and motor delays, autism, intellectual disability, and sleep disturbances [23].

Intriguingly, BRAT1 (BRCA1-associated ATM activator 1), a direct binding partner of INTS11 [24–26], is also mutated in a spectrum of neurological disorders, including *nonprogressive cerebellar ataxia* (NPCA), *BRAT1-related rigidity and multifocal seizure syndrome* (RMFSL) and *BRAT1-related neurodevelopmental disorder with cerebellar atrophy and with or without seizures syndrome* (NEDCAS) [27, 28]. Despite well-established clinical links, the molecular mechanisms underlying BRAT1-associated disease heterogeneity and the physiological functions of BRAT1 remain poorly understood.

Considering the clinical overlap between BRAT1- and INTS11-related disorders, together with evidence supporting BRAT1’s role in Integrator complex integrity [24–26], we hypothesized a shared molecular aetiology. In particular, we sought to determine whether disorders associated with *INTS11* and *BRAT1* mutations converge on a common defect in RNA processing. To address this, we aimed to expand the mutational spectrum of *INTS11* and *BRAT1* and to systematically asses the functional impact of these variants, with a focus on U snRNA processing, particularly U1 snRNA. In addition, we set out to establish an in vivo zebrafish model of *ints11* deficiency to investigate the molecular and neurodevelopmental consequences of Integrator dysfunction. Finally, we explored the potential of unprocessed U1 snRNA as a biomarker for disease classification and patient stratification. Collectively, this work aims to define whether defects in U1 snRNAs processing represent a shared molecular signature of BRAT1- and INTS11-related disorders and to provide a framework for diagnostic and translational applications.

## Methods

### Patient ascertainment, clinical and genetic evaluation

**Fig. 1:**
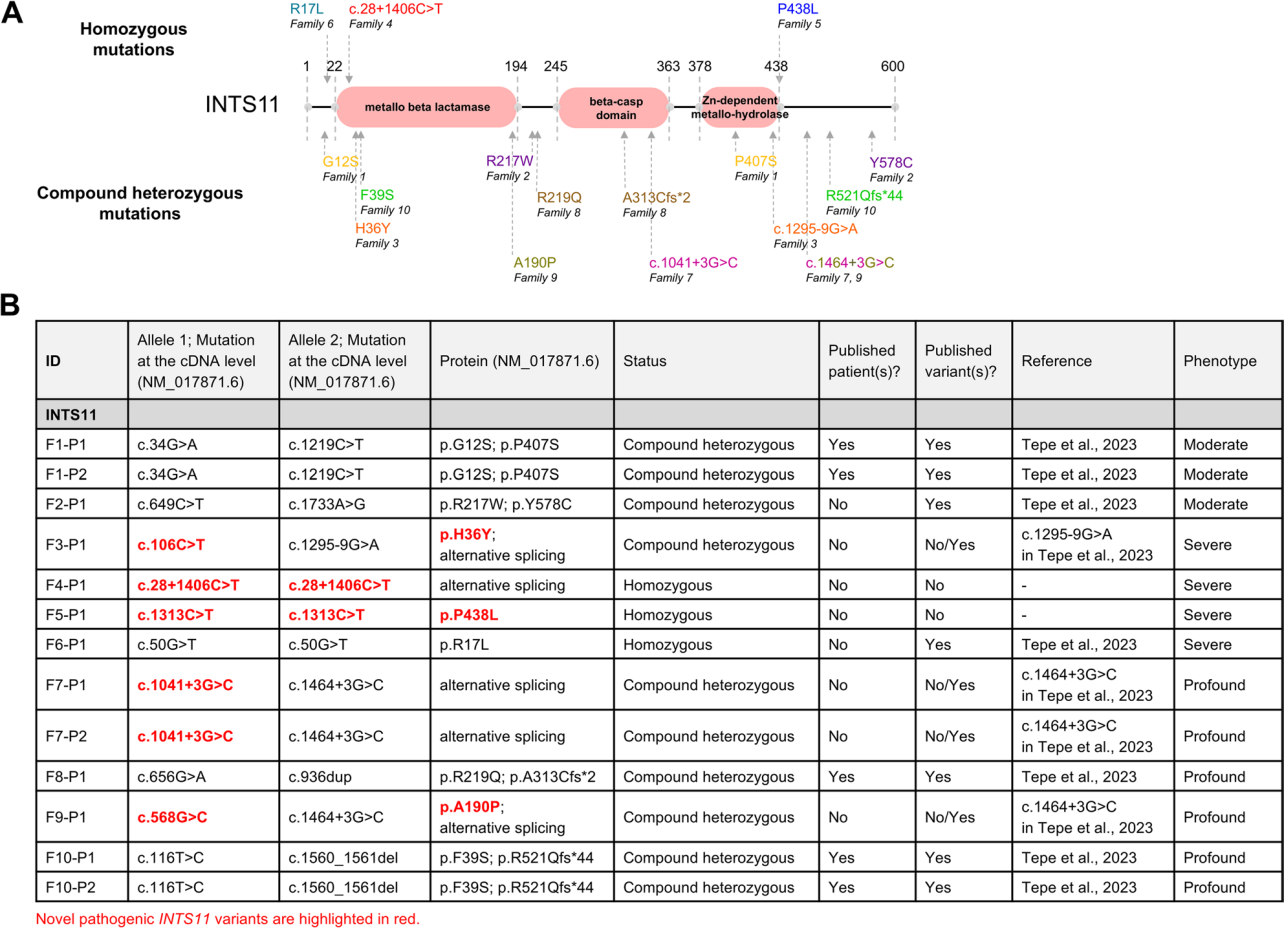
INTS11 structure and variants examined in this study. **A** Schematic representation of INTS11 mutations analysed in this study, spanning the entire gene. The variants include two frameshift mutations leading to premature stop codons, ten missense mutations, and four splicing-affecting mutations. Variants present in compound heterozygous patients are color-coded accordingly. **B** Summary of INTS11-mutated patients analysed in this study. The summary includes the cDNA and protein-level annotations of each variant, along with their homozygous or compound heterozygous status. Clinical severity for each patient, categorized as moderate, severe, or profound, is also indicated

**Fig. 2:**
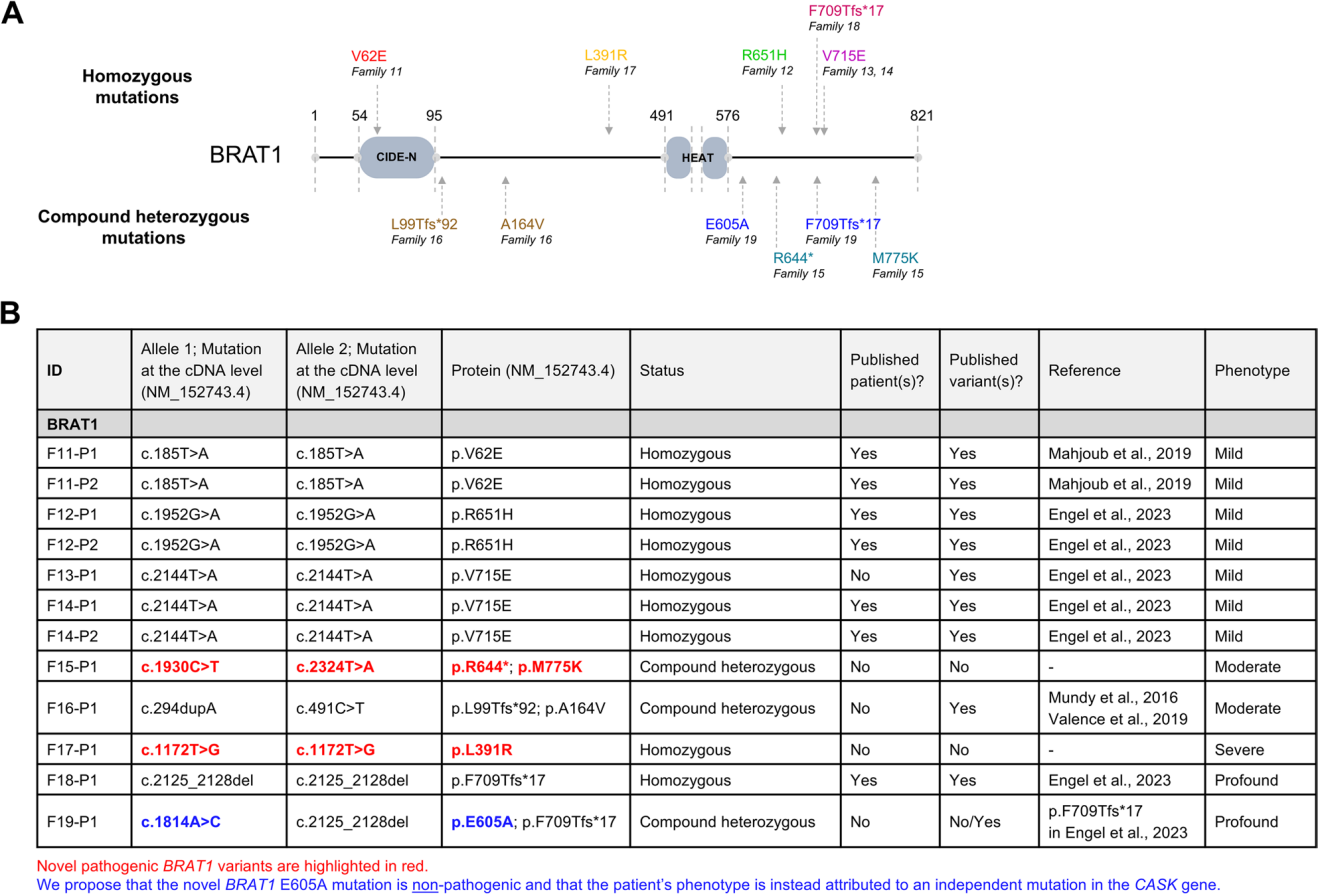
BRAT1 structure and variants examined in this study. **A** Schematic representation of BRAT1 mutations analysed in this study, spanning the entire gene. The variants include two frameshift mutations resulting in premature stop codons, seven missense mutations, and one missense variant that introduces a premature stop codon. Variants present in compound heterozygous individuals are color-coded accordingly. **B** Summary of BRAT1-mutated patients analysed in this study. Mutations are annotated at both the cDNA and protein levels, along with their homozygous or compound heterozygous status. Clinical severity of each case, ranging from mild to profound, is also provided

Affected individuals with biallelic *INTS11* and *BRAT1* mutations were identified through collaboration, systematic screening of diagnostic and research genetic databases worldwide, and submissions to GeneMatcher [29]. Informed consent for both diagnostic and research-based studies was obtained from all participants and/or their legal guardians, in accordance with local ethical guidelines. Whole-exome or genome sequencing was performed on genomic DNA extracted from peripheral blood in various diagnostic or research laboratories. Where applicable, candidate variants were confirmed by Sanger sequencing in probands and available family members. Clinical data were systematically collected for both newly recruited and previously reported cases by contributing clinicians and were independently reviewed and analysed (Additional file 1: Fig. S1A, B; Fig. S2A, B). The clinical parameters assessed included global developmental delay (GDD) and intellectual disability (ID), as well as neurological features such as ambulation status, spasticity, ataxia, seizures, microcephaly and brain imaging abnormalities. We analysed the clinical data of 13 patients with biallelic *INTS11* mutations (*n* = 13), including three previously reported cases with updated follow-up, two published cases, and eight novel cases (Fig. 1A, B; Additional file 1: Fig. S1A, B). In parallel, we analysed 12 individuals with biallelic BRAT1 mutations (*n* = 12), comprising seven previously reported and five novel cases (Fig. 2A, B; Additional file 1: Fig. S2A, B). The cohort included both male and female patients.

### Cell lines and culture conditions

This study employed patient-derived primary fibroblasts and lymphoblastoid cell lines (LCLs) obtained from individuals carrying biallelic variants in *INTS11* or *BRAT1* (Figs. 1B and 2B). Cells from unaffected heterozygous parents were included where available and are referred to as “*parents*”, not as controls. Unrelated control samples consisted of one primary fibroblast line (*n* = 1; *denoted* 1BR ctrl) and one LCL line (*n* = 1; *denoted* LCL ctrl), as previously described [30]. For *INTS11*, primary fibroblasts were obtained from nine patients with biallelic variants (*n* = 9) and four unaffected parents (*n* = 4) carrying a single *INTS11* mutation. LCLs were established from one affected individual (*n* = 1) and both parents (*n* = 2). For *BRAT1*, primary fibroblasts were obtained from eight individuals with biallelic variants (*n* = 8) and four unaffected parents (*n* = 4) carrying a single *BRAT1* mutation. LCLs were available from three affected individuals (*n* = 3) and four parents (*n* = 4). The sex of all patient individuals is indicated in the corresponding figures (Additional file 1: Fig. S1A; Fig. S2A). Information on the sex of some parent samples was not available. Written informed consent was obtained at the time of sample collection for the derivation of cell lines and their use in research. Human fibroblasts were cultured in Minimum Essential Medium Eagle (MEM; Gibco) supplemented with 15% fetal bovine serum (FBS; Gibco), 2 mM L-glutamine (Gibco), and antibiotics (100 units/ml penicillin and 100 µg/ml streptomycin; Gibco) at 37 °C in a humidified atmosphere with 5% CO_2_. LCLs were maintained in Roswell Park Memorial Institute 1640 Medium (RPMI 1640; Sigma) supplemented with 10% FBS and the antibiotics (100 units/ml penicillin and 100 µg/ml streptomycin; Gibco), under identical conditions.

### Generation of *ints11-KO* zebrafish

Multiple independent studies have demonstrated that CRISPant phenotyping is robust when carefully designed and validated [31–34]. Accordingly, we generated *ints11* F0 knockouts zebrafish (*ints11-KO*; CRISPants) using clustered regularly interspaced short palindromic repeats/Cas9 (CRISPR/Cas9) system. Three single-guide RNAs (gRNAs) were designed using the online tool CRISPRscan (protospacer adjacent motif in parentheses), targeting exon 4: CAGGATGGGGCAGATGGCTT(TGG), exon 9: CCCGGTGGAGAAATAAATGG(GGG), and exon 13: AGCGCTCACGCAGACGCCAA(AGG) of the *ints11* gene. Control gRNAs were designed similarly with five mismatches in the 3’ region of their gene-specific sequences (mismatches in lowercase): exon 4: CAGGAUGGGGCcGgUUaCcU, exon 9: CCCGGUGGAGAgAcAUAgGU, and exon 13: AGCGCUCACGCcGgCUCUAg. In-vitro transcribed Cas9 mRNA and synthetic 2’MOE-modified gRNAs (Synthego) were microinjected into one-cell-stage embryos as described previously [35]. For each assay, the mutagenic score of the gRNAs and the non-mutagenicity of 5-mismatch gRNAs were confirmed by high-resolution melting genotyping [35].

### Zebrafish husbandry and care

All animal experiments were conducted at the University of Montreal Hospital Center (CHUM) Research Center in accordance with institutional animal care guidelines and the Canadian Council for Animal Care. Adult zebrafish (*Danio rerio*) were maintained at 28.5 °C under a 12-hour light/dark cycle and staged as described previously [36]. Embryos were obtained by natural spawning and raised in E3 medium at 28.5 °C. Larvae were monitored daily for morphology, behaviour, and survival, and no unexpected adverse phenotypes requiring early humane endpoints were observed under control conditions. When required, larvae were euthanized by overdose of tricaine methanesulfonate (MS-222), followed by confirmation of death in accordance with Canadian Council on Animal Care guidelines.

### Phenotypic characterization in zebrafish

Morphological analysis was performed on larvae at 5 days post-fertilization (dpf). Larvae were immobilized in a 3% methylcellulose cavity, and images were captured using a Leica S6E stereomicroscope. Body length, head size, and eye size were measured from scale-calibrated images using ImageJ software (National Institutes of Health, Bethesda, Maryland).

### Behaviour assay in zebrafish

The swimming behaviour of 5 dpf larvae was monitored using DanioVision (Noldus Wageningen, The Netherlands) during a 1-hour dark period followed by a 1-hour light period. Larvae were separated into single wells of a 96-well plate containing 200 µL of E3 media and habituated in the Daniovision recording chamber for 1 h before the experiment. Ethovision XT12 (Noldus) software was used to analyse swimming distance and maximum acceleration during the recorded periods.

### Antibodies

Primary antibodies used in this study were as follows: anti-BRAT1 C-terminal (WB 1:50.000; Abcam, ab181855), anti-BRAT1 (WB 1:1000; Thermo Fisher Sc., PA530916), anti-INTS11 (WB 1:1000; Novus Biologicals, NB100-60638), anti-INTS9 (WB 1:1000; Cell Signalling, 13945), anti-INTS4 (WB 1:1000; Abcam, ab75253), anti-β-actin (WB 1:5000; Protein Tech, 66009), anti-α-tubulin (WB 1:8000; Abcam, ab6160), anti-α-tubulin (WB 1:1000; Santa Cruz, sc-23948), anti-Parvalbumin7 (immunostaining of zebrafish larvae 1:1000, mouse monoclonal, gift from Dr Hibi, Nagoya University, Japan). Secondary antibodies employed for western blotting were HRP-conjugated goat anti-rabbit (1:10.000; Bio-Rad, 170–6515), goat anti-mouse (1:10.000; Bio-Rad, 170–6516) and rabbit anti-rat (1:10.000, Abcam, ab6734).

### Immunostaining of zebrafish larvae

*ints11* larvae were fixed in 4% paraformaldehyde in PBS for 1 h at room temperature. Following gradual rehydration in PBST, the larvae were permeabilized for 10 min in ice-cold acetone. Blocking and permeabilization were performed using 5% normal goat serum, 1% BSA and 1% DMSO in 1X PBS for 1.5 h at room temperature. The larvae were then incubated overnight at 4 °C with anti-Parvalbumin7 antibody (gift from Dr. Hibi, Nagoya U., Japan). After several washes, they were incubated overnight at 4 °C with a goat anti-mouse secondary antibody conjugated to Alexa Fluor 488. Fluorescence was analysed using confocal microscopy and images were processed with ImageJ software (National Institutes of Health, Bethesda, Maryland).

### Total RNA extraction and RT-qPCR analysis

Total RNA was extracted from 5 dpf whole larvae using the PicoPure RNA extraction kit (Thermo Fisher Scientific) following the manufacturer’s standard protocol. For each sample, RNA was isolated from a pool of five whole larvae. RNA quality was assessed using a Nanodrop spectrophotometer, measuring the 260/280 and 260/230 ratios to check for potential chemical contamination. Reverse transcription was performed using 500 ng of total RNA with the Superscript VILO Reverse Transcription Mix (Invitrogen). Quantitative reverse-transcription PCR (RT-qPCR) was conducted on a LightCycler 96 (Roche) using 2 µL of 1:10-diluted cDNA, SYBR Green I Master (Roche), and the following target primer pairs:

- U1_4snRNA_F-AAATGTGGGAATCTCGACTGCATG and
- U1_4snRNA_R-CTCGTGTGTCCTTGATTGTGTGTG (U1 snRNA unprocessed);
- U2_snRNA_F-GCATCGACCCGGTATTGCAG and
- U2_snRNA_R-ACGAACCAATCTCCACATGC (U2 snRNA unprocessed);
- U2_1snRNA_F-GCATCGACCCGGTATTGCAG and
- U2_1snRNA_R-TCGTTGATACACATCATTCG (U2_1 snRNA unprocessed);
- U2_2snRNA_F-GCATCGACCCGGTATTGCAG and
- U2_2snRNA_R-ATGTATCAATCCGTCTTATCTC (U2_2 snRNA unprocessed);
- U5_snRNA_F-TGATGCCCTGCCTATCGGTG and
- U5_snRNA_R-AGGTTCCATCCGTTATTTCTCTTTC (U5 snRNA unprocessed);
- U6_1_snRNA_F-GCAAACCCGTTAAGCGATCCAT and
- U6_1_snRNA_R-CACCTCCCACGATTTGCTAAAC (U6_1 snRNA unprocessed);
- U6_2_snRNA_F-GCAAATTCGTGAAGCGTTCCTC and
- U6_2_snRNA_R-TTTCCCTCACCGGATACTGT (U6_2 snRNA unprocessed);
- U6_3_snRNA_F-ACGCAAATTCGTGAAGCGTTCC and
- U6_3_snRNA_R-CATTGCACGCTCAAACTCGGT (U6_3 snRNA unprocessed);
- U6_4_snRNA_F-CGCAAATTCGTGAAGCGTTCCA and
- U6_4_snRNA_R-GGATGCGCTGCGTACTGAAC (U6_4 snRNA unprocessed);
- U12_snRNA_F-TTTGAACGGGTACAGGTCTGC and
- U12_snRNA_R-TTTAACCTGTTATTGGGTGTTGTCG (U12 snRNA unprocessed);
- ef1a_F-GTGGCTGGAGACAGCAAGA and
- ef1a_R-AGAGATCTGACCAGGGTGGTT (Elongation Factor 1 α);
- gapdh_F-TTGAGAAACCTGCCAAGTATGA and
- gapdh_R-CCCATTGAAGTCAGTGGACA (Glyceraldehyde-3-phosphate dehydrogenase);
- polr2d_F-AACGCAAAGTGGGAGATGTG and
- polr2d_R-AGCGTCTCTGCGTTCTCAA (RNA polymerase II subunit D)

For human control, unaffected parents and patient-derived cells, total RNA was extracted using the RNeasy Mini Kit with an additional Dnase I digestion (Qiagen; 74104 and 79254) following the manufacturer’s instructions. RNA quality was assessed using Nanodrop spectrophotometry. One microgram of total RNA was reverse transcribed into cDNA using RevertAid Reverse Transcriptase (Life Technologies) with random hexamer primers. Quantitative reverse-transcription PCR (RT-qPCR) was performed on a LightCycler 480 (Roche) using SYBR Green PCR Master Mix (Life Technologies) and the following target primer pairs:

- RNU1_F-GAAACTCGACTGCATAATTTGTGGTAG and
- RNU1_R-CTTGGCGTACAGTCTGTTTTTGAAACTC (U1 snRNA unprocessed);
- RNU2_F-AACATAGGTACACGTGTGCCACGG and
- RNU2_R-ACAAATAGCCAACGCATGCGGGGC (U2 snRNA unprocessed);
- RPLP2_F-TCTTGGACAGCGTGGGTATCGA and
- RPLP2_R-CAGCAGGTACACTGGCAAGCTT (Large Ribosomal Subunit Protein P2);
- ACTB_F-CACCATTGGCAATGAGCGGTTC and
- ACTB_R-AGGTCTTTGCGGATGTCCACGT (Actin β).

The expression data were normalized to reference genes expression levels, and relative expression was calculated by Pffafl method:

$$\:\mathrm{R}\mathrm{Q}=\frac{{2}^{{\Delta\:}\mathrm{C}\mathrm{t}\left(\mathrm{t}\mathrm{a}\mathrm{r}\mathrm{g}\mathrm{e}\mathrm{t}\right)}}{{2}^{{\Delta\:}\mathrm{C}\mathrm{t}\left(\mathrm{r}\mathrm{e}\mathrm{f}\mathrm{e}\mathrm{r}\mathrm{e}\mathrm{n}\mathrm{c}\mathrm{e}\right)}}$$

### Characterisation of alternative splicing

The mutated region of the *INTS11* transcript, containing the mutation c.28 + 1406 C > T, was amplified from isolated cDNA using DreamTaq polymerase (Thermo Fisher, EP0702) with the following primers: INTS11exon1_F-ATCAGAGTCACGCCCTTG and INTS11exon4_R-ACCATCTCGCTGAAGTAG. The alternatively spliced variant was separated by agarose gel electrophoresis and extracted using QIAquick Gel Extraction Kit (BioTech, 28704). The purified PCR product containing the patient mutation was inserted into a TOPO TA vector following the manufacturer’s instructions (TOPO™ TA Cloning™ Kit, Invitrogen, 450641). The presence of alternative exon usage was identified by Sanger sequencing using the following primers: M13_F-GTAAAACGACGGCCAG and M13_R-CAGGAAACAGCTATGAC.

To detect the alternative splicing caused by *INTS11* intronic mutations c.1041 + 3G > C/c.1464 + 3G > C and c.1295-9G > A, cDNA was amplified using DreamTaq Green PCR Master Mix (Thermo Fisher, K1082) with the following primers:

- INTS11exon9_1F-CCACTACTACAAGCTGTTC, INTS11exon9_2F-CCAGAAGATCCGCAAGAC, and INTS11exon16_2R-CACACAGTGGTCCTTCAG (Tm 60 °C, 2% gel; GeneRuler 1Kb Plus DNA Ladder and SM0241) or
- INTS11exon9_1F-CCACTACTACAAGCTGTTC, INTS11exon16_1R-ACAGTCACAGAGCCGTCTGG, and INTS11exon16_2R-CACACAGTGGTCCTTCAG (Tm 62 °C, 3% gel; GeneRuler 100 bp Plus DNA Ladder), respectively.

### SDS-PAGE and western blotting

Cells were harvested and lysed in SDS sample buffer (2% SDS, 10% glycerol, 50 mM Tris-HCl, pH 6.8), denatured at 95 °C for 10 min, and sonicated with a Bioruptor^®^ Pico (Diagenode) for 30 s. Protein concentrations were determined using the BCA assay (Pierce; 23227). DTT and bromophenol blue were added to the samples, which were then resolved by SDS-PAGE. Proteins were transferred to a nitrocellulose membrane and detected with the appropriate primary antibody in combination with a horseradish peroxidase-conjugated secondary antibody. Peroxidase activity was induced and detected using ECL reagent (GE Healthcare) and Amersham Hyperfilm ECL (GE Healthcare). The exposed film was scanned, and the digital image was analysed using Image Studio Lite version 5.2 software (LI-COR Biosciences). The intensity of the signal was normalized against the loading control (β-actin or α-tubulin).

### Fluorescence in situ hybridization (FISH)

Cells cultured on glass coverslips were fixed in 4% formaldehyde for 10 min, permeabilized by 70% ethanol for 1 hour on ice, and washed three times with PBS. Hybridization was then performed using a fluorescent DNA probe (BIOSEARCH technologies #SMF-1083-5, Ref. #SS847331-01-30) according to the manufacturer’s instructions (Stellaris RNA-FISH; LGC Biosearch Technologies; SMF-WA1-60, SMF-HB1-10, SMF-WB1-20 and SMF-1083-5). The fluorescently labelled DNA probe was designed to specifically anneal to the unprocessed region of U1 snRNA, spanning approximately 1000 bp downstream from 3’ box. After hybridization, the samples were washed in PBS, and nuclei were stained with DAPI. Coverslips were mounted using Vectashield anti-fading mounting reagent (Vector Laboratories). Fluorescence was analysed and images were acquired using a Leica DM6000 microscope. For each genotype, at least 121 cells were quantified (*n* ≥ 121) across two independent biological replicates. The number of FISH foci and their intensity were quantified by *CellProfiler 4.2.5*. Nuclei were identified using the *IdentifyPrimaryObjects* segmentation module with global two-class Otsu thresholding, applying a threshold factor of 0.95 and a threshold smoothing scale of 1.3488. Objects with diameters between 50 and 400 pixels were retained. Foci were detected using the *IdentifyPrimaryObjects* segmentation module with global thresholding based on the *Robust Background* method, applying a threshold factor of 0.95 and a threshold smoothing scale of 1.3488. The lower and upper outlier fractions were set to 0.05. Objects with diameters between 3 and 20 pixels were retained. For both nuclei and foci, objects touching the borders were excluded.

### Statistical analysis

Statistical analyses of zebrafish data were performed using GraphPad Prism 10. At least ten individual samples, gathered from at least three independent clutches were used for each assay. Statistical analyses of the patient data were performed using the XLMiner Analysis ToolPak add-in for Microsoft Excel and GraphPad Prism 10. Statistical comparisons were performed as follows. For small sample sizes (*n* ≤ 4), two-tailed Student’s *t*-tests were used to analyse the data and assess significance across experimental replicates, with confidence intervals calculated at the 95% level. For larger sample sizes (*n* ≥ 5), normality was first assessed using the Shapiro-Wilk test. Data that met normality assumptions were analysed using two-tailed Student’s *t*-tests, whereas data that did not meet these assumptions were analysed using the non-parametric Mann-Whitney test. The statistical test applied to each comparison is indicated in the corresponding figure legend. Results were considered statistically significant when *p*-value was < 0.05. Statistical significance levels are indicated by asterisks as follows: (*), (**), (***), and (****) corresponding to *p*-value of < 0.05, 0.01, 0.001, and 0.0001, respectively.

## Results

### Characterization of patients with biallelic *INTS11* mutations

Biallelic *INTS11* variants have previously been associated with neurodevelopmental disorders in 18 patients from 12 unrelated families [20–22]. We expand this spectrum by identifying five novel homozygous or compound heterozygous variants and characterizing 13 affected individuals (7 males, 6 females) from 10 unrelated families (F1-10; Fig. 1A, B; Additional file 1: Fig. S1A, B). Three cases had been previously reported and are presented here with longitudinal follow-up data, two are published cases, while the remaining eight are novel [21]. Age at last evaluation ranged from 6 months to 20 years. Parental consanguinity was reported in 38% (5/13) of the cohort. At the time of data collection, 77% (10/13) of the individuals were alive. INTS11-related disease was consistently associated with global developmental delay (GDD), intellectual disability (ID), microcephaly, and brain imaging abnormalities. Variably present features included ataxia and spasticity. We classified clinical severity into moderate, severe, and profound phenotypic groups based on the presence and extent of developmental delay, intellectual capacity, mortality, and the severity of neurological features.

Moderate Phenotype (*n* = 3; mean age of 16.3 years at last follow-up – range: 10–20 years).

Patients in this group presented in infancy, and all exhibited moderate GDD/ID. Ambulation was achieved with support, with first steps at a mean age of 3.5 years (range: 2–5 years), and all were able to speak a few words. Microcephaly and ataxia were present in all cases. Spasticity was noted in one individual. Seizures occurred in one patient, with adolescent onset, generalised tonic-clonic in nature, and response to treatment. Neuroimaging revealed cerebellar atrophy in all case, with additional findings including pontocerebellar atrophy (1/3) and diffuse white matter changes (1/3).

Severe Phenotype (*n* = 4; mean age of 4.8 years at last follow-up – range: 1–8 years).

Symptom onset occurred in the neonatal period in half the cases (2/4) and in infancy in the other half (2/4). All individuals exhibited severe GDD/ID. Two individuals achieved ambulation with support (at 2.5 and 5 years), and one was able to speak a few words. Microcephaly was present in 3/4, ataxia in 2/4, spasticity in 1/4, and one individual experienced seizures (1/4). Neuroimaging revealed cerebellar atrophy in all individuals (4/4), a thin corpus callosum in two (2/4), and periventricular dystrophy with delayed myelination in one (1/4).

Profound Phenotype (*n* = 6; mean age of 4.9 years at last follow-up – range: 0.5–18 years).

All profoundly affected individuals presented in the prenatal or neonatal period. They showed profound GDD/ID with complete absence of ambulation or speech. The half of the individuals (3/6) were deceased at the time of data collection. Microcephaly was observed in all individuals (6/6), and spasticity was more frequent in this subgroup (3/6). All experienced seizures, typically with early onset and poor response to treatment. Neuroimaging revealed cerebral and cerebellar atrophy in most cases (4/5), pontocerebellar atrophy (2/5), gyral simplification (2/6), leukoencephalopathy and severe hypomyelination (2/6), and a thin corpus callosum (1/6).

### Characteristics of *BRAT1*-mutated patients and overlap with INTS11-related disease

Our previous studies have identified a physical interaction between INTS11 and BRAT1 (BRCA1-associated ATM activator 1), a protein implicated in a spectrum of neurological disorders ranging from mild neurodegeneration to severe neurodevelopmental syndromes [24, 28, 37]. To further investigate a functional connection between the genes and disease, we systematically analysed the clinical phenotypes of patients with biallelic *BRAT1* mutations, including four novel variants, and compared them with those of individuals harbouring pathogenic *INTS11* variants. We evaluated 12 individuals (7 males, 5 females) from nine unrelated families (F11-19) including seven previously reported and five newly described cases (Fig. 2A, B; Additional file 1: Fig. S2A, B) [28, 38]. Age at last clinical evaluation ranged from 5 months to 24 years, and 92% (11/12) of the individuals were alive at the time of data collection. Parental consanguinity was reported in 44% (4/9) of families. The core phenotype associated with BRAT1-related disorder includes early-onset GDD, ID, and brain MRI abnormalities, with variable presence of ataxia, spasticity, seizures, and microcephaly. To further delineate the phenotype, affected individuals were categorised into four groups: mild, moderate, severe, and profound, based on mortality, the presence and extent of developmental delay, intellectual capacity, and the severity of neurological features.

Mild Phenotypes (*n* = 7; mean age of 7.5 years at last follow-up – range: 3–24 years).

Symptom onset occurred during infancy in all evaluated cases. All individuals achieved ambulation, with first steps at a mean age of 1.9 years (range: 1.3–2.3 years), although most of them required support. All individuals experienced delays in speech development and exhibited mild ID. Ataxia was present in all cases, while spasticity was reported in one individual (1/7). None of the mildly affected individuals displayed seizures or microcephaly. Neuroimaging revealed cerebellar atrophy in all and corpus callosum atrophy in 2/7.

Moderate Phenotypes (*n* = 2; mean age of 5 years at last follow-up).

Symptom onset occurred during the prenatal period or infancy. Both individuals achieved ambulation, with 1/2 requiring support. Speech development was limited to a few words, and both exhibited moderate ID. One individual (F15-P1) displayed spasticity and seizures, while the other (F16-P1) presented with ataxia. Microcephaly was absent. Neuroimaging demonstrated cerebellar atrophy in both cases.

Severe phenotype (*n* = 1).

Individual F17-P1 presented with a severe phenotype, with onset during the first year of life. She was unable to walk, her language capacity was limited to two words, and displayed severe ID. She started to experience early-onset seizures at the age of 2 months. Clinical examination revealed microcephaly, hypotonia, and ataxia. Neuroimaging revealed cerebral and cerebellar atrophy, cerebellar white matter changes, corpus callosum hypoplasia, and a simplified gyral pattern.

Profound Phenotypes (*n* = 2; mean age of 1.5 years at last follow-up).

This subgroup presented within the first month of life. All individuals exhibited profound GDD, microcephaly, and seizures typically beginning within the first year; one was refractory to treatment. One individual (F18-P1) showed limb spasticity with axial hypotonia, while ataxia was absent in both cases. Neuroimaging revealed cerebral atrophy in one individual and cerebellar atrophy in the other. One individual (F18-P1) died at 14 months due to seizure-related complications.

### Impact of *INTS11* variants on protein level and Integrator function in patient-derived cells

The *INTS11* variants span the entire coding region (NM_017871.6), including two frameshifts, ten missense, and four predicted splice-disrupting mutations (Fig. 1A; Additional file 1: Fig. S1B) [39]. To examine their effects, we established cell cultures from skin biopsies or blood samples of patients, as well as from unaffected heterozygous parents carrying a single *INTS11* mutation. Primary fibroblasts were obtained from nine different patients: three homozygous (*denoted* R17L^HOM^, c.28 + 1406 C> T^HOM^, and P438L^HOM^) and six compound heterozygous (*denoted* G12S/P407S, H36Y/c.1295-9G > A, F39S/R521Qfs*44, A109P/c.1464 + 3G > C, R217W/Y578C, and c.1041 + 3G > C/c.1464 + 3G > C). We collected samples from four unaffected parents (*denoted* c.28 + 1406 C> T^HET^, A109P^HET^, P438L^HET^ and c.1464 + 3G> C^HET^) and an unrelated healthy individual (*denoted* 1BR ctrl). Additionally, lymphoblastoid cell lines (LCLs) were generated from one compound heterozygous patient (*denoted* R219Q/A313Cfs*2), the patient’s unaffected parents (*denoted* R219Q^HET^ and A313Cfs*2^HET^) and an unrelated control (*denoted* LCL ctrl).

**Fig. 3:**
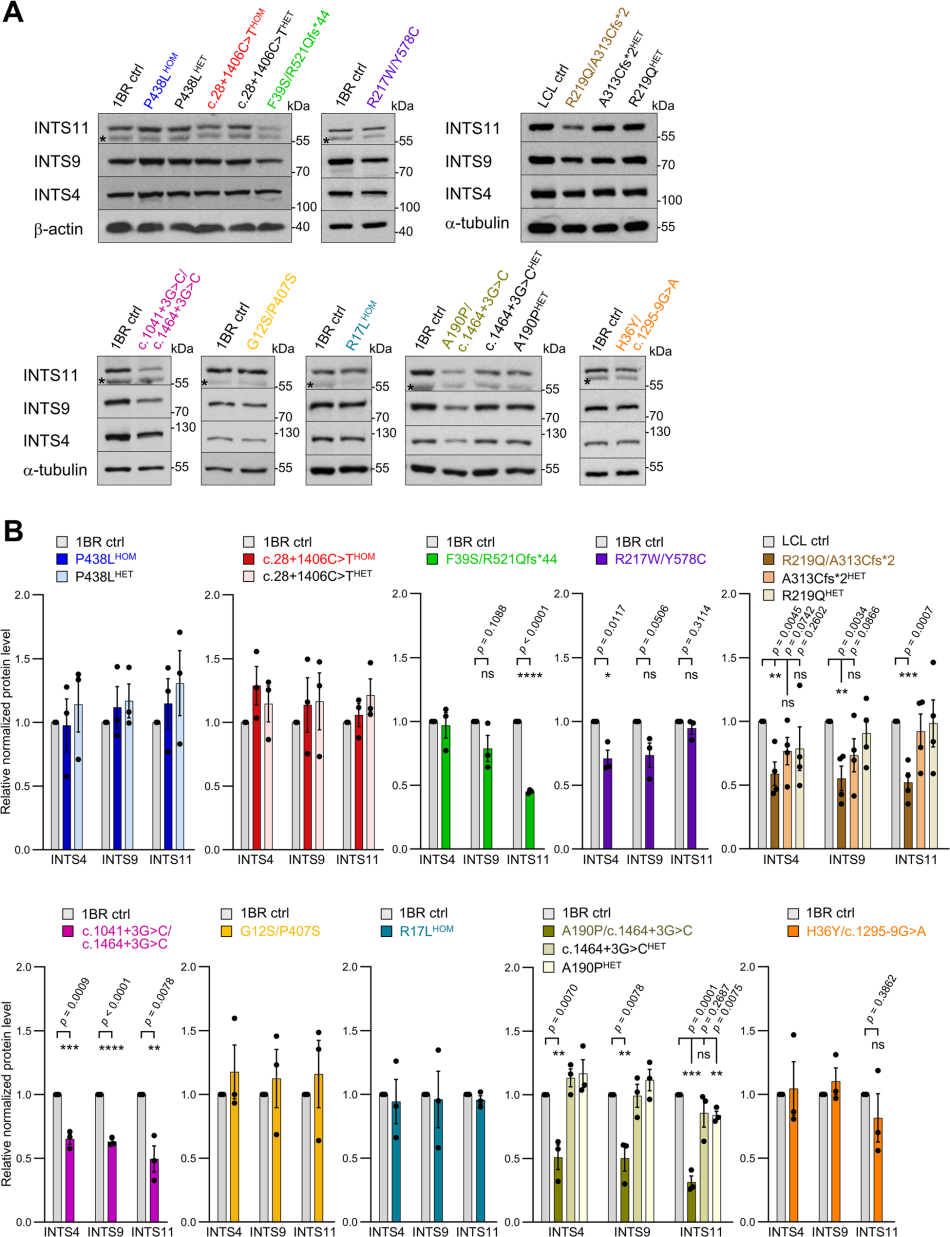
Analysis of Integrator subunit protein levels in *INTS11*-mutated patient-derived cells. **A** Immunoblot analysis of INTS11, INTS9, and INTS4 protein levels in fibroblasts and lymphoblastoid cell lines (LCLs) derived from the healthy control, unaffected parents or INTS11-mutated patients. β-actin or α-tubulin was used as a loading control. A non-specific band is indicated by an asterisk (*). **B** Quantification of protein levels from (A), based on at least three independent experiments. Data are presented as mean ± SEM. Statistical significance was determined using a two-tailed Student’s t-test (ns – not significant, *p < 0.05, **p < 0.01, ***p < 0.001, ****p < 0.0001)

To assess the impact of these mutations on INTS11 abundance, we first compared protein levels across patients, unaffected parents and a healthy control. We found that INTS11 levels were markedly reduced only in cell lines derived from four patients with compound heterozygous mutations F39S/R521Qfs*44, A109P/c.1464 + 3G > C, R219Q/A313Cfs*2 and c.1041 + 3G > C/c.1464 + 3G > C (Fig. 3A, B). This suggests that these mutations directly impair the formation or stability of the full-length INTS11 protein. Additionally, two other Integrator subunits, INTS9 and INTS4, which interact with INTS11 to form the core catalytic cleavage module, were significantly reduced in three of these patients (Fig. 3A, B), indicating compromised complex stability.

Next, to evaluate the impact of intronic mutations on splicing efficiency, we amplified *INTS11* transcripts from isolated cDNA of three relevant patients, unaffected parents and control cells, as outlined in Additional file 1: Fig. S3A. Specifically, the c.1041 + 3G > C/c.1464 + 3G > C variants in introns 10 and 14 promoted retention of these introns, or both, in the mature mRNA (Additional file 1: Fig. S3B). The c.28 + 1406 C > T variant, located in the first intron, promoted the usage of an alternative exon, generating a distinct transcript variant (Additional file 1: Fig. S3C, E). The alternative transcript was detected in the unaffected c.28 + 1406 C> T^HET^ parent but was far more abundant in the c.28 + 1406 C> T^HOM^ patient and absent in the unrelated 1BR control, where only the canonical isoform is present. The canonical transcript (ENST00000435064.6) encodes a protein composed of 600 amino acids, while the alternatively spliced transcript (ENST00000545578.5) uses an alternative start codon in exon 3, resulting in a shorter protein of 571 amino acids (Additional file 1: Fig. S3A). We propose that this shorter protein may be non-functional, as it lacks the first 29 amino acids, including part of the metallo-beta-lactamase domain (Fig. 1A). Additionally, the c.1295-9G > A variant in intron 12 promoted splicing changes leading to a frameshift mutation (Additional file 1: Fig. S3D). The canonical spliced transcript is still detected, which could correspond to the splicing of the allele carrying the H36Y missense mutation and to incomplete penetrance of the c.1295-9G > A variant. Together, these findings suggest that while some variants directly reduce INTS11 protein levels, missense mutations or altered splicing may impair protein function or interactions, potentially altering Integrator cleavage complex activity.

**Fig. 4:**
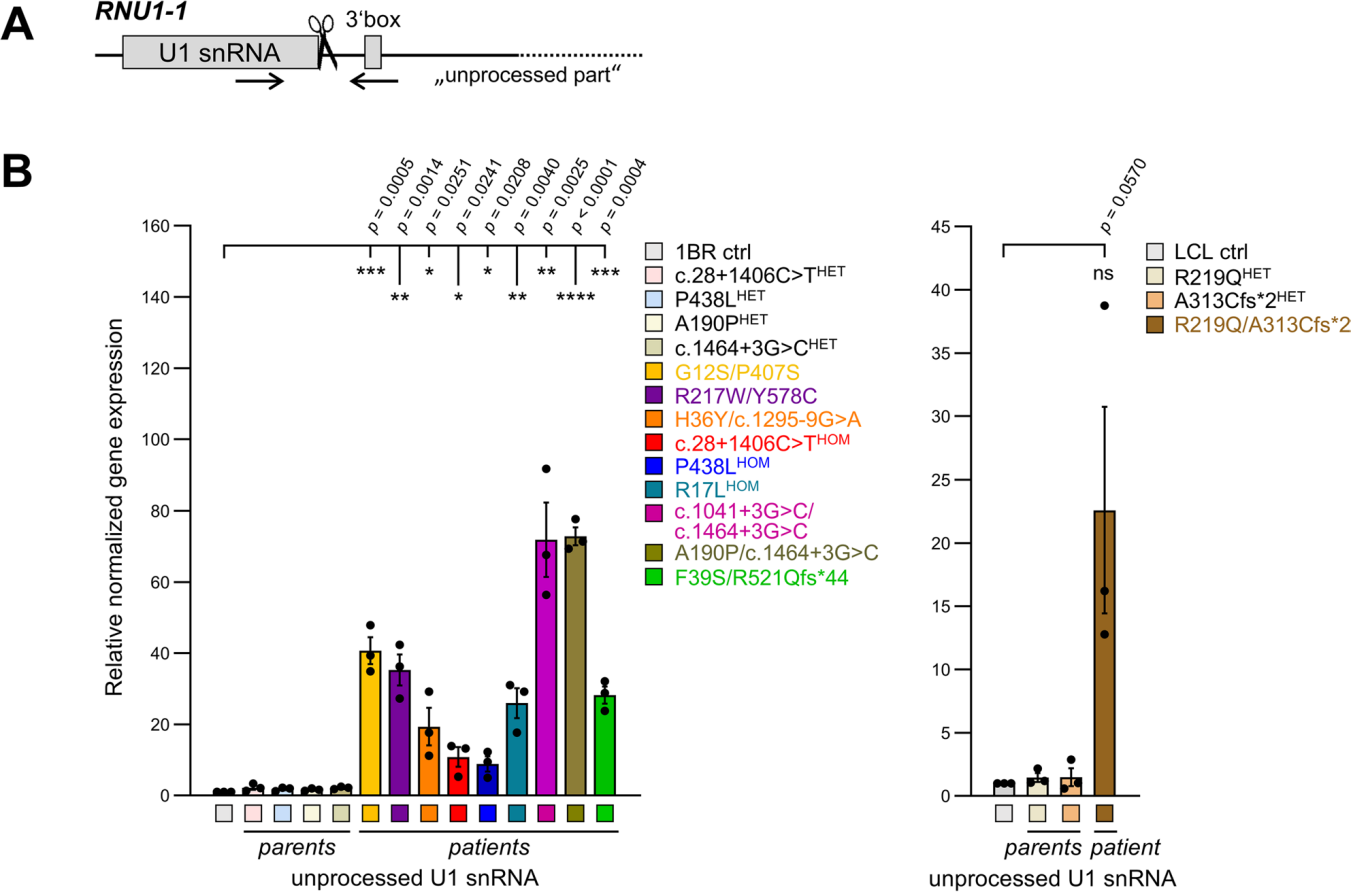
Impaired 3′-end processing of U1 snRNAs in *INTS11*-mutated patient-derived cells. **A** Schematic representation of the RT-qPCR approach used to detect unprocessed U1 snRNAs transcribed from the RNU1-1 gene. The diagram indicates the positions of RT-qPCR primers (arrows), the canonical Integrator-mediated cleavage site (scissors), and the downstream 3′ box. **B** RT-qPCR analysis of unprocessed RNU1-1 transcripts in control, parent and INTS11-mutated patient-derived fibroblasts (left) and LCLs (right). Data are presented as mean ± SEM from three independent experiments (*N* = 3). Statistical significance was determined using a two-tailed Student’s t-test (ns – not significant, *p < 0.05, **p < 0.01, ***p < 0.001, ****p < 0.0001)

To evaluate functional consequences of various biallelic *INTS11* mutations, we analysed the accumulation of unprocessed U snRNAs across different patient-derived cell lines using quantitative reverse-transcription PCR (RT-qPCR). Primers targeting unprocessed U1 and U2 snRNAs encoded by the human *RNU1-1* and *RNU2-1* genes (Fig. 4A; Additional file 1: Fig. S4A), previously validated in siINTS11-depleted cells [24], revealed a pronounced increase in long unprocessed forms of U snRNAs, particularly U1 snRNAs, in all patient samples, even in those with unchanged INTS11 protein levels (Fig. 4B; Additional file 1: Fig. S4B). The most striking defects were observed in patients with the profound clinical phenotype carrying complex-destabilizing mutations (A109P/c.1464 + 3G > C, R219Q/A313Cfs*2, and c.1041 + 3G > C/c.1464 + 3G > C). These findings underscore the critical role of Integrator in efficient snRNA 3’-end processing and suggest that unprocessed U snRNAs, particularly U1, may serve as a sensitive readout for pathogenic *INTS11* variants.

### *Ints11*-deficient zebrafish exhibit impaired U snRNA processing and recapitulate human neurodevelopmental deficits

**Fig. 5:**
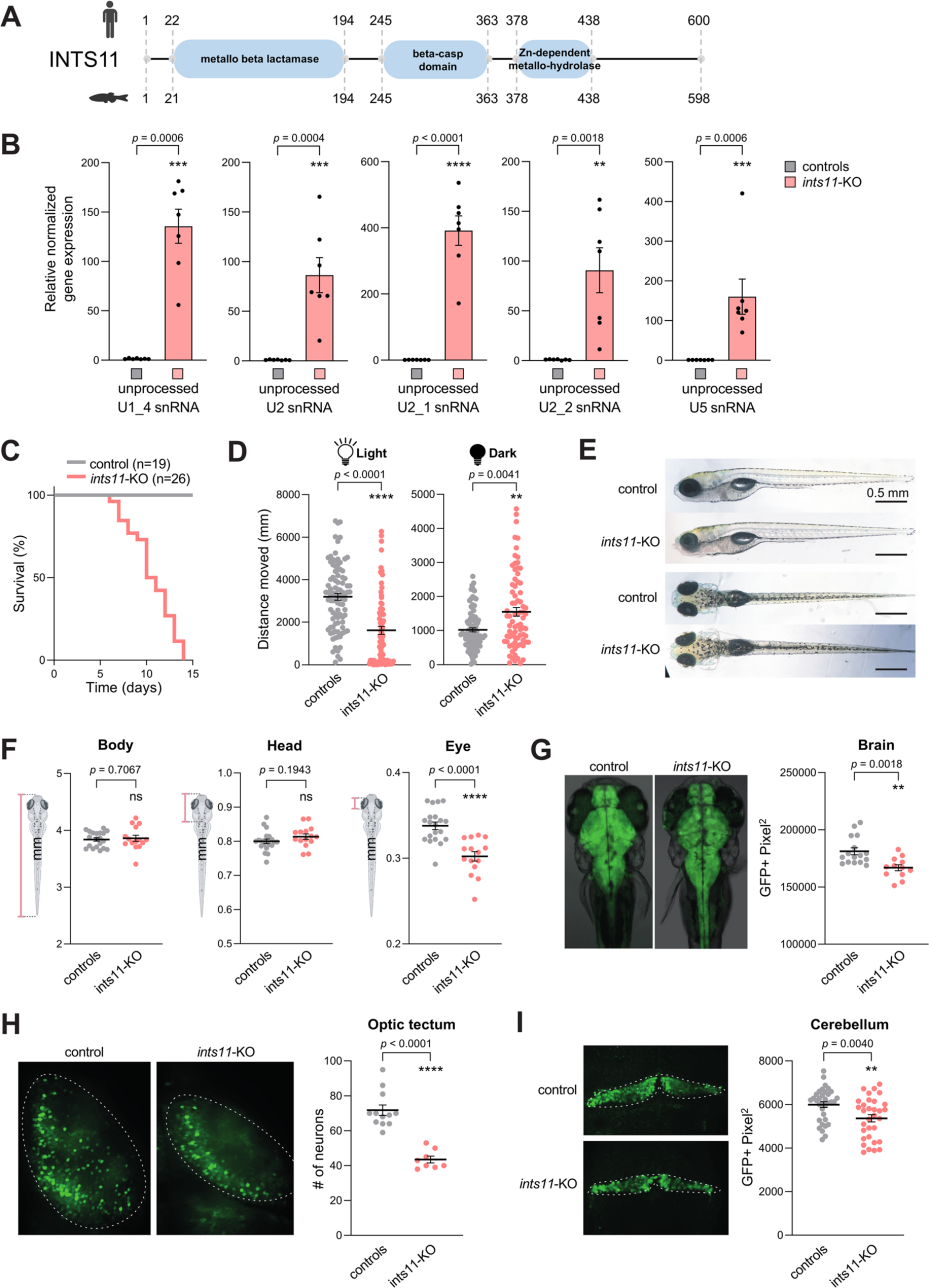
*ints11*-deleted zebrafish exhibit increased unprocessed U snRNAs, abnormal motor behaviour, and reduced brain size. **A** Schematic representation of the three main INTS11 functional domains (metallo-beta-lactamase, beta-CASP, and C-terminal) which are conserved between human and zebrafish. **B** RT-qPCR analysis of 3’ unprocessed snRNAs (U1_4, U2, U2_1, U2_2, U5) in 5 days post-fertilization (dpf) larvae. Data show fold-change relative to controls (ΔΔCt method). *P*-values from the Mann-Whitney test for U1_4 and U5; *p*-values from the two-tailed Student’s t-test for U2, U2_1 and U2_2: **p < 0.01, ***p < 0.001, ****p < 0.0001 (n > 35, *N* = 3). **C** Kaplan-Meier survival analysis over 14 dpf comparing ints11-KO larvae (*n* = 26) with controls (*n* = 19). **D** Quantification of total distance moved (mm) per larvae over one hour using an automated recording chamber tracking. The total distance swam by ints11-KO larvae was significantly increased in the dark (**p < 0.01) but decreased in the light (****p < 0.0001) compared to controls. *P*-values from the Mann-Whitney test; sample sizes: controls (*n* = 93), ints11-KO (*n* = 77). **E** Representative bright-field lateral and dorsal images of control and ints11-KO larvae at 5 dpf. Scale bar: 0.5 mm. **F** Quantification of body size, head length, and eye diameter in control (*n* = 20) and ints11-KO (*n* = 16) larvae. *P*-values from the two-tailed Student’s t-test: ****p < 0.0001; ns – not significant. **G** Representative dorsal images of 5 dpf larval brains from Tg(elavl3: GFP) transgenic lines and quantification of brain size showing a significant reduction in ints11-KO larvae. *P*-value from the Mann-Whitney test: **p < 0.01; sample sizes: controls (*n* = 18), ints11-KO (*n* = 13). **H** Confocal imaging (20×) of Tg(elavl3:GFP) larvae at 5 dpf revealing a significant reduction in neuronal content in ints11-KO larvae compared to controls. *P*-value from the two-tailed Student’s t-test: ****p < 0.0001; sample sizes: controls (*n* = 12), ints11-KO (*n* = 8). **I** Confocal images (20×) of 5 dpf larvae immunolabeled with anti-parvalbumin-7 (PAV7), revealing a significant reduction in cerebellar size in ints11-KO larvae compared to controls. *P*-value from the two-tailed Student’s t-test: **p < 0.01; sample sizes: controls (*n* = 33), ints11-KO (*n* = 34)

Since INTS11-related disease has only recently been described, we developed a zebrafish knockout model (*ints11*-KO) to investigate the functional consequences of *INTS11* deficiency in vivo, with particular focus on the brain. Zebrafish possess a single ortholog of human *INTS11* (*ints11*, ENSDARG00000025212), which encodes a protein sharing 84% sequence identity with the human INTS11 and retains all functionally annotated domains (Fig. 5A). Using CRISPR/Cas9 mutagenesis, we generated F0 CRISPants by targeting three exons encoding these conserved domains (exon 4, 9, and 13). Embryos injected with the same mutagenic tools but containing 5-mismatch control gRNAs were used as controls (*see* Methods). The mutagenic efficiency for *ints11* and mismatch control gRNAs was confirmed using high-resolution melting genotyping (Additional file 1: Fig. S5A). To assess the functional impact of *ints11* deletion levels on U snRNA processing, we quantified unprocessed U snRNAs in *ints11*-KO larvae using RT-qPCR with primers targeting the 3’ unprocessed regions of zebrafish snRNAs (*U1_4*,* U2*,* U2_1*,* U2_2*,* U5*, *U6_1*,* U6_2*,* U6_3*,* U6_4* and *U12*). Our analysis revealed a significant accumulation of unprocessed U1, U2, U5 and U12 snRNAs in 5-day-old *ints11*-KO larvae compared to mismatch controls (Fig. 5B; Additional file 1: Fig. S5B). In contrast, U6 snRNA processing in the zebrafish *ints11-KO* CRISPants was largely unaffected (Additional file 1: Fig. S5B), consistent with the established role of the Integrator complex in RNA polymerase II–dependent snRNA transcription [2, 40]. These results confirm the functional consequences of disruption of *ints11* and are in agreement with previous findings in *Drosophila*, *C. elegans*, and mammalian cells, confirming that INTS11 loss selectively impairs processing of both major and minor spliceosomal snRNAs [41–44].

Strikingly, *ints11*-KO larvae exhibited premature lethality, failing to survive beyond 14 days post-fertilization (dpf) (Fig. 5C). Prior to death, affected larvae displayed significant behavioural abnormalities. Specifically, *ints11*-KO larvae showed sustained dark-time hyperactivity and generalized light-time hypoactivity when assessed at 5 dpf using a dark-light cycle assay (Fig. 5D; Additional file 1: Fig. S5C). These behavioural changes resemble those observed in zebrafish models of other neurodevelopmental disorders [45–47]. Notably, the severity of the hypoactivity phenotype correlated with the mutagenic score of *ints11*-targeting gRNAs, confirming the specificity of the phenotype (Additional file 1: Fig. S5D). Despite no overt morphologic abnormalities at 5 dpf, *ints11*-KO larvae exhibited a significant reduction in eye diameter, suggesting potential effects on the central nervous system (Fig. 5E, F). To further investigate neurodevelopmental deficits, we estimated brain size in *ints11*-KO larvae using the Tg[elavl3:GFP] transgenic line, which expresses green fluorescent protein (GFP) in post-mitotic neurons [48]. We observed a significant reduction in brain size compared to controls (Fig. 5G), most notably in the midbrain, which was associated with a notable decrease in neuronal content in the optic tectum, the largest brain structure (Fig. 5H; Additional file 1: Fig. S5E). While the overall hindbrain size appeared unaffected, cerebellar integrity was compromised. Immunostaining of Purkinje cells using an anti-parvalbumin-7 (PAV7) antibody revealed a significant reduction in cerebellar size, as estimated by PAV7 + staining (Fig. 5I).

Together, these findings establish *ints11*-KO zebrafish as a powerful in vivo model of INTS11 deficiency. The constellation of phenotypes, including impaired U snRNA processing, abnormal behaviour, reduced brain and cerebellar volume, and early lethality, mirrors the clinical manifestations observed in *INTS11*-mutated patients. These data further underscore the critical and conserved role of INTS11 in vertebrate brain development and function.

### *BRAT1* mutations destabilize INTS11 and disrupt the Integrator cleavage complex

**Fig. 6:**
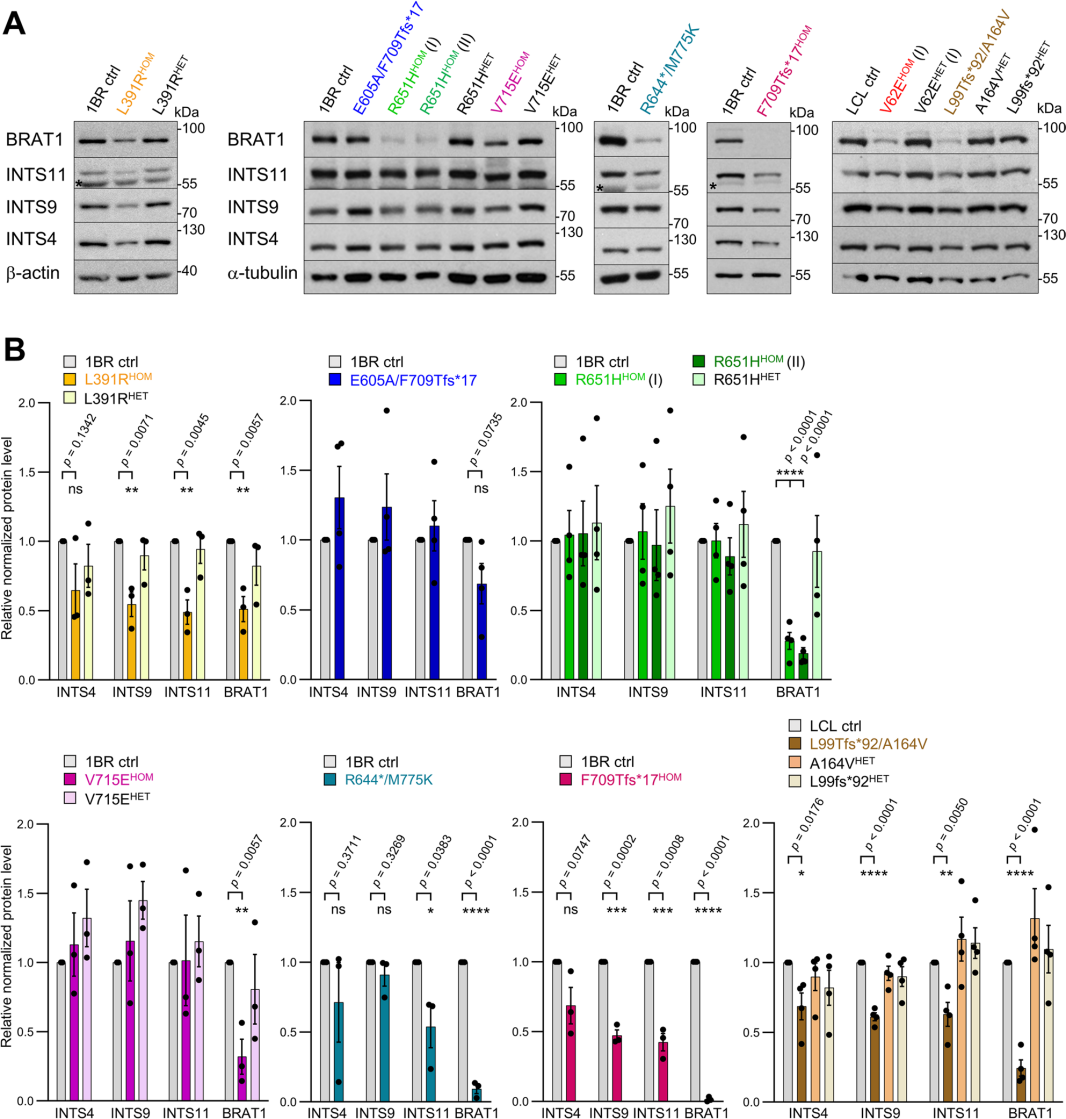
Analysis of BRAT1 and Integrator subunit protein levels in *BRAT1*-mutated patient-derived cells. **A** Immunoblot analysis of BRAT1, INTS11, INTS9, and INTS4 in fibroblasts and lymphoblastoid cell lines (LCLs) derived from the healthy control, unaffected parents and BRAT1-mutated patients. β-actin or α-tubulin was used as a loading control. A non-specific band is indicated by an asterisk (*). **B** Quantification of protein levels presented in (A) from at least three independent experiments. Data are shown as the mean ± SEM. Statistical significance was determined using a two-tailed Student’s t-test (ns – not significant, *p < 0.05, **p < 0.01, ***p < 0.001, ****p < 0.0001)

To test whether *BRAT1* mutations impair INTS11 stability and Integrator function, we established primary fibroblasts from six individuals with homozygous biallelic *BRAT1* mutations (*denoted* V62E^HOM^, L391R^HOM^, R651H^HOM^(I), R651H^HOM^(II), F709TFs*17^HOM^, and V715E^HOM^) and from two individuals with compound heterozygous mutations (*denoted* E605A/F709Tfs*17 and R644*/M775K). We also included fibroblasts from four unaffected parents carrying a single *BRAT1* mutation (*denoted* V62E^HET^(I), L391R^HET^, R651H^HET^, and V715E^HET^) and used 1BR cells as controls. Additionally, we employed lymphoblastoid cell lines (LCLs) from two affected siblings with homozygous *BRAT1* mutations (*denoted* V62E^HOM^(I) and V62E^HOM^(II)), one patient with compound heterozygous mutations (*denoted* L99Tfs*92/A164V), and their unaffected parents (*denoted* V62E^HET^(I), V62E^HET^(II), L99Tfs*92^HET^, and A164V^HET^). Immunoblot analysis revealed consistently reduced BRAT1 protein levels across all patient samples, except in fibroblasts from the compound heterozygous individual E605A/F709Tfs*17 (Fig. 6A, B). Notably, cells from patients carrying the L391R^HOM^, R644*/M775K, F709Tfs*17^HOM^, and L99Tfs*92/A164V mutations, similarly to the previously studied V62E^HOM^ cells, exhibited a marked reduction in INTS11 protein levels [24]. In particular, cells from the L391R^HOM^ and F709Tfs*17^HOM^ patients also showed decreased INTS9 levels, while the L99Tfs*92/A164V cells displayed a reduction in the entire Integrator cleavage complex (Fig. 6A, B). These findings provide strong evidence that BRAT1 is required for maintaining INTS11 stability and the structural integrity of the Integrator cleavage complex, consistent with recent structural studies [25, 26].

### Nuclear accumulation of 3’ unprocessed U1 snRNAs in *INTS11*- and *BRAT1*-mutated patient cells

**Fig. 7:**
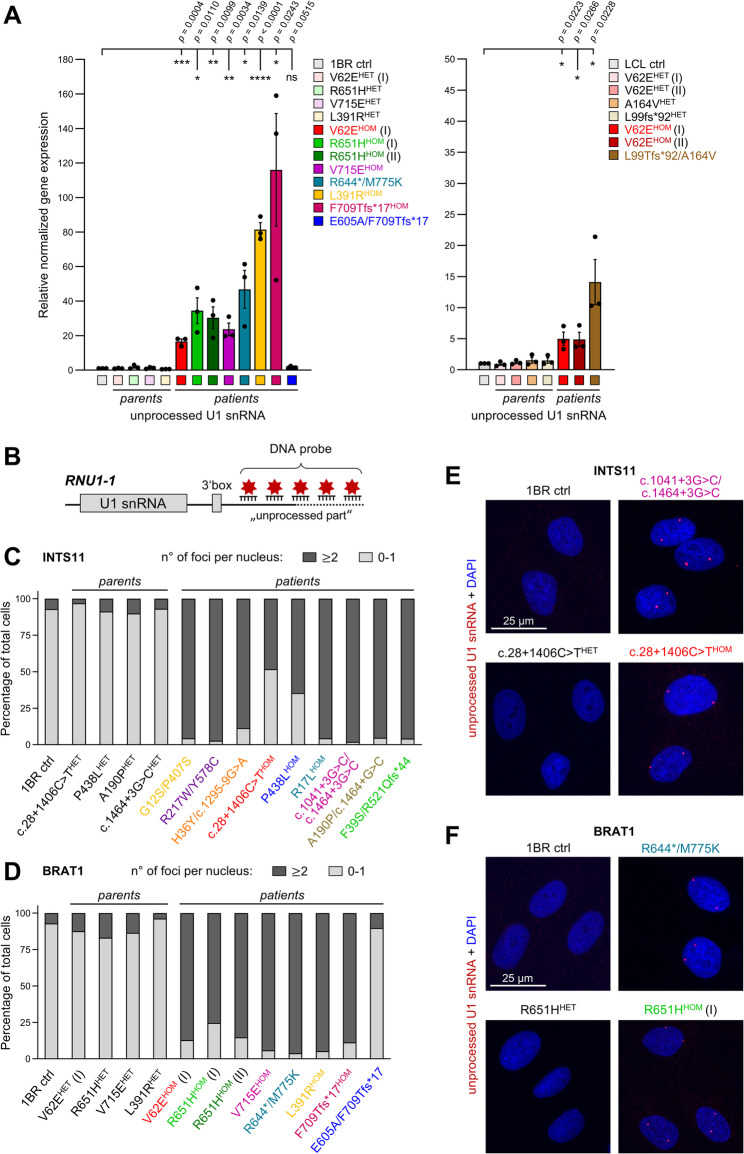
Nuclear accumulation of 3’-extended unprocessed U1 snRNAs in patient-derived cells with *BRAT1* and *INTS11* mutations. **A** RT-qPCR analysis of unprocessed RNU1-1 transcripts in control, parent and BRAT1-mutated patient-derived fibroblasts (left) and LCLs (right). Data are presented as the mean ± SEM (*N* = 3; *N* = 4 for E605A/F709Tfs*17 patient). Statistical significance was calculated using a two-tailed Student’s t-test (ns – not significant, *p < 0.05, **p < 0.01, ***p < 0.001, ****p < 0.0001). **B** Schematic representation of the FISH strategy used to detect 3’-extended unprocessed RNU1-1 transcripts. The DNA probe anneals to the unprocessed region of U1 snRNA approximately 1000 bp downstream of the canonical 3’ box. **C**-**D** Quantification of FISH-detected unprocessed U1 snRNA foci in INTS11-mutated (**C**) and BRAT1-mutated (**D**) fibroblasts. Results are expressed as the percentage of nuclei containing U1 foci. For each genotype, at least 121 cells were quantified (*n* ≥ 121) across two independent biological replicates. **E**-**F** Representative FISH images of INTS11-mutated (**E**) or BRAT1-mutated (**F**) fibroblasts, along with the 1BR control and unaffected parents. Unprocessed U1 snRNAs are visualized using specific fluorescent probes (red), while nuclei are stained with DAPI (blue). Scale bar: 25 μm

To determine whether *BRAT1* mutations impair Integrator-mediated U snRNA processing, we analysed 3’-end processing of U snRNAs in patient-derived cells. Using RT-qPCR with primer sets specific for the unprocessed forms of U snRNAs encoded by the human *RNU1-1* and *RNU2-1* genes, we detected a significant accumulation of 3’ unprocessed U1 snRNAs in nearly all patient samples (Fig. 7A). A notable exception was observed for fibroblasts from the patient with E605A/F709Tfs*17 compound heterozygous variants. Interestingly, in addition to showing no significant changes in BRAT1 protein levels, this patient did not exhibit substantial increase in unprocessed U1 snRNAs, suggesting that the novel E605A variant may be non-pathogenic. Notably, this individual also carries a likely pathogenic variant in *CASK* (Calcium/calmodulin-dependent Serine protein Kinase), which could account for the profound neurological phenotype (Fig. 2B; Additional file 1: Fig. S2A). In contrast, analysis of U2 snRNA processing revealed only a modest increase in unprocessed transcripts that did not reach statistical significance, although a similar trend toward accumulation was observed in several patient samples (Additional file 1: Fig. S4C). This finding illustrates the diagnostic utility of U1, the most abundant snRNA in human cells, and its processing assays for functional classification of uncertain BRAT1 variants. Excitingly, the most severe U1 snRNA processing defects were observed in individuals carrying homozygous F709Tfs*17 or L391R mutations. With the notable exception of the E605A/F709Tfs*17 patient, the degree of U1 snRNA misprocessing closely correlated with clinical severity, supporting the potential of defective U1 snRNA processing as a biomarker for BRAT1-related disease.

To further investigate the fate of misprocessed U1 snRNAs in patient cells, we performed fluorescence in situ hybridization (FISH) using a DNA probe complementary to the unprocessed region of U1 snRNA located approximately 1000 bp downstream of the 3’ box (Fig. 7B). This approach revealed nuclear accumulation of long, unprocessed U1 snRNAs in cells from patients with both *INTS11* and *BRAT1* mutations, with the exception of fibroblasts from the E605A/F709Tfs*17 individual (Fig. 7C-F; Additional file 1: Fig. S6A-C). Notably, these unprocessed U1 snRNAs localized to discrete nuclear foci, likely corresponding to U1 transcription sites, as indicated by the presence of two distinct signals per nucleus representing both alleles [49, 50]. These findings suggest that improperly processed U1 snRNAs are not rapidly degraded but instead accumulate in the nucleus, where they may interfere with essential cellular processes. Together, these results confirm that defective U1 snRNA 3’-end processing is a hallmark of INTS11- and BRAT1-related disease and underscore its utility as a molecular readout for disease severity. Furthermore, the presence of nuclear U1 snRNA accumulation could serve as a reliable biomarker for assessing the pathogenicity of novel *INTS11* and *BRAT1* variants, thereby aiding in the diagnosis, classification of variants, and stratification of clinical risk in affected individuals.

## Discussion

The Integrator complex is essential for embryogenesis and cell differentiation, with loss of its subunits, including the catalytic endonuclease INTS11, resulting in lethality across species [1]. Here, we establish a zebrafish model that confirms this conserved requirement. *Ints11*-KO larvae failed to survive beyond 14 days post-fertilization and exhibited profound neurodevelopmental abnormalities, including reduced brain size, decreased neuronal content, and aberrant behaviours. Specifically, *ints11*-KO larvae displayed hypoactivity in light and hyperactivity in darkness, a pattern reminiscent of behavioural phenotypes reported in other zebrafish models of neurodevelopmental disorders [45–47]. These findings parallel the larval lethality described in Drosophila *dInts11* mutants and position zebrafish as a vertebrate system for modelling INTS11 dysfunction [21]. Importantly, loss of INTS11 in zebrafish resulted in marked accumulation of unprocessed major and minor spliceosomal U snRNAs, confirming its conserved role in RNA 3’-end processing [2, 42, 43]. This disruption recapitulates key molecular and neurodevelopmental features observed in patients carrying *INTS11* mutations, further underscoring its relevance for human disease [20–22].

Although pathogenic *INTS11* variants have been recently linked to neurodevelopmental disorders, the impact on RNA metabolism in patient-derived cells remained poorly defined. Only one prior study examined patient cells, describing a single variant (D106N) impairing INTS11 catalytic activity [22]. By analysing a broader cohort of individuals carrying distinct *INTS11* variants (Fig. 1A), we uncover a shared defect in U1 snRNA 3’-end processing across all patients. Importantly, these defects occurred even in cells with near-normal INTS11 protein levels, indicating that many pathogenic variants disrupt Integrator function without affecting protein levels. This points to multiple mechanisms of pathogenicity, including impaired catalytic activity, defective INTS9/INTS11 heterodimer assembly, or altered incorporation into the larger Integrator complex.

Building on our previous discovery that BRAT1 interacts with the INTS9/INTS11 heterodimer [24], and recent studies identifying BRAT1 as a chaperone of the Integrator cleavage module [25, 26, 51], we extended our analyses to patient cells carrying *BRAT1* mutations. Consistent with impaired Integrator function, mutations spanning the entire *BRAT1* gene (Fig. 2A) led to the elevated levels of unprocessed U1 snRNAs, with the severity of misprocessing correlating with clinical outcome, providing the first evidence of a genotype-to-phenotype relationship in BRAT1-associated disease. Notably, while the use of a single unrelated control represents a limitation, all heterozygous parents consistently exhibited very low levels of unprocessed U1 snRNAs, supporting the conclusion that the observed defects are primarily driven by biallelic variants. The most pronounced defects were observed in the patient homozygous for the F709Tfs*17 mutation, whereas moderate defects were associated with the V62E variant and other mutations linked to milder phenotypes. Importantly, the compound heterozygous E605A/F709Tfs*17 patient exhibited near-normal BRAT1 protein levels and no measurable U1 snRNA misprocessing, strongly suggesting that the E605A variant is benign. Given the presence of a concurrent likely pathogenic variant in *CASK*, we propose that the neurological phenotype in this patient is primarily CASK-driven [52]. These findings highlight the diagnostic potential of U1 snRNA processing assays for evaluating uncertain *BRAT1* variants, with direct implications for genetic counselling and patient management. Furthermore, considering the recent identification of WDR73 as another INTS9/INTS11 interactor that modulates cleavage function [25, 53], we suggest that U1 snRNA processing analysis could be extended to neurological disorders caused by *WDR73* mutations.

Collectively, our data establish impaired RNA processing as a core molecular hallmark of both INTS11- and BRAT1*-*related disorders. Pathogenic variants disrupt Integrator activity through distinct mechanisms, including direct catalytic impairment, defective complex assembly, or loss of regulatory partners, but they converge on the same biochemical endpoint, the nuclear accumulation of unprocessed U1 snRNAs. Interestingly, consistent with previous observations from our laboratory and others, mature U snRNA levels and global splicing appear largely preserved upon Integrator depletion [2, 19, 24, 54]. Moreover, the unprocessed U1 snRNAs persist in discrete nuclear foci, suggesting they are not rapidly degraded and may interfere with genome stability, RNA processing, or transcriptional homeostasis. These effects may be particularly deleterious in post-mitotic neurons, ultimately contributing to neuronal dysfunction and degeneration. Importantly, Integrator complex has functions beyond U snRNA processing. INTS11 participates in the cleavage of diverse RNA species, including both protein-coding and noncoding RNAs. Integrator modulates gene expression via premature transcription termination, and defects in this pathway have been linked to activation of the integrated stress response (ISR) through accumulation of double-stranded RNA originating from intron-retaining transcripts [55]. Together, disruption of these processes may collectively contribute to the disease pathology observed in patients with Integrator dysfunction [56]. Understanding these broader consequences of Integrator dysfunction provides important insights into disease pathogenesis and may inform the development of diagnostics approaches and potential therapeutic strategies.

## Conclusions

This study establishes impaired U1 snRNA 3′-end processing as a shared molecular hallmark of INTS11- and BRAT1-related neurological disorders. It provides direct evidence that *BRAT1* mutations disrupt U1 snRNA processing, clarifying its functional role and broadening the spectrum of Integrator-related pathology. Nuclear accumulation of unprocessed U1 snRNAs emerges as a biologically grounded biomarker for variant interpretation, disease severity assessment, and patient stratification, particularly in BRAT1-associated cases with previously uncharacterized functional consequences. Integration of patient-derived cell analyses with a zebrafish model of *ints11* deficiency validates the in vivo impact of Integrator dysfunction and offers a mechanistic framework for understanding neuronal vulnerability. Collectively, these findings yield insight into the molecular aetiology of these disorders and provide a foundation for improved diagnostics and translational research in RNA-processing–associated neurodevelopmental disease.

## Supplementary Information

Additional file 1. Supplementary figures: Fig. S1: Clinical overview and variants of INTS11-mutated patients. Fig. S2: Clinical overview and variants of BRAT1-mutated patients. Fig S3. Affected splicing in patients with INTS11 intronic mutations. Fig. S4: 3’-end processing of U2 snRNAs in INTS11- and BRAT1-mutated patient cells. Fig. S5: Characterization of the ints11-deleted zebrafish model. Fig. S6: Nuclear accumulation of unprocessed U1 snRNAs in BRAT1- and INTS11-mutated patient-derived cells.

Additional file 2. Uncropped blots and electrophoretic gels: Related to Fig. 3: Uncropped western blots for evaluation of INTS11, INTS9 and INTS4 protein levels in INTS11-mutated patients. Related to Fig. 6: Uncropped western blots for evaluation of BRAT1, INTS11, INTS9 and INTS4 protein levels in BRAT1-mutated patients. Related to Fig. S3B-D: Uncropped electrophoretic gels for evaluation of alternative splicing in patients with INTS11 intronic mutations.

## Data Availability

All data generated or associated with this study are included in this published article and its Supplementary Information files.

## Abbreviations

snRNAs: Small nuclear RNAs
INTS11: Integrator complex subunit 11
RNAPII: RNA polymerase II
eRNAs: Enhancer RNAs
lncRNAs: Long-noncoding RNAs
piRNAs: PIWI-interacting RNAs
ID: Intellectual disability
BRAT1: BRCA1-associated ATM activator 1
NPCA: Nonprogressive cerebellar ataxia
RMFSL: Rigidity and multifocal seizure syndrome
NEDCAS: Neurodevelopmental disorder with cerebellar atrophy and with or without seizures syndrome
GDD: Global developmental delay
RT-qPCR: Quantitative reverse-transcription PCR
dpf: Days post-fertilization
GFP: Green fluorescent protein
PAV7: Parvalbulim-7
LCLs: Lymphoblastoid cell lines
CASK: Calcium/calmodulin-dependent serine protein kinase
FISH: Fluorescence in situ hybridization
ISR: Integrated stress response
MEM: Minimum Essential Medium
FBS: Fetal bovine serum
RPMI: Roswell Park Memorial Institute 1640 Medium
gRNAs: Single-guide RNAs
