# Supplementary material for: Unprocessed U1 snRNAs as a biomarker of INTS11- and BRAT1-related neurodevelopmental disorders
Source: Genome Med. 2026 May 12;18:95. doi: 10.1186/s13073-026-01667-1 (PMC13335351; doi:10.1186/s13073-026-01667-1)
Supplement: Supplementary file 1 — Additional file 1. Supplementary figures: Fig. S1: Clinical overview and variants of INTS11-mutated patients. Fig. S2: Clinical overview and variants of BRAT1-mutated patients. Fig S3. Affected splicing in patients with INTS11 intronic mutations. Fig. S4: 3’-end processing of U2 snRNAs in INTS11- and BRAT1-mutated patient cells. Fig. S5: Characterization of the ints11-deleted zebrafish model. Fig. S6: Nuclear accumulation of unprocessed U1 snRNAs in BRAT1- and INTS11-mutated patient-derived cells. [file 13073_2026_1667_MOESM1_ESM.pdf]

Additional file 1

Unprocessed U1 snRNAs as a biomarker of INTS11- and BRAT1-related neurodevelopmental disorders

A

Gene	Participant ID	c-DNA	Protein	Sex/Cons.	Age f/u	GDD/ID	Ambulation	Spasticity	Ataxia	Seizures	Microcephaly	Abn. MRI	Phenotype
INTS11	F1-P1[a]	c.34G>A; c.1219C>T	p.G12S; p.P407S	M / -	20 y	++	+	-	+	+	+	+	moderate
	F1-P2[b]	c.34G>A; c.1219C>T	p.G12S; p.P407S	F / -	19 y	++	+	-	+	-	+	+	moderate
	F2-P1	c.649C>T; c.1733A>G	p.R217W; p.Y578C	F / -	10 y	++	+	+	+	-	+	+	moderate
	F3-P1	c.106C>T; c.1295-9G>A	p.H36Y; p.?	M / -	8 y	+++	-	+	-	-	+	+	severe
	F4-P1	c.28+1406C>T	p.?	F / +	6 y	+++	+	-	+	-	+	+	severe
	F5-P1	c.1313C>T	p.P438L	M / +	4 y	+++	+	-	+	-	-	+	severe
	F6-P1	c.50G>T	p.R17L	F / +	1 y	+++	-	-	-	+	+	+	severe
	F7-P1	c.1041+3G>C; c.1464+3G>C	p.?	M / +	1 y†	+++	-	+	-	+	+	+	profound
	F7-P2	c.1041+3G>C; c.1464+3G>C	p.?	M / +	2 y	+++	-	+	-	+	+	NA	profound
	F8-P1[c]	c.656G>A; c.936dup	p.R219Q; p.A313Cfs*2	M / -	2 y	+++	-	+	NA	+	+	+	profound
	F9-P1	c.568G>C; c.1464+3G>C	p.A190P; p.?	M / -	6 mo	+++	-	-	NA	+	+	+	profound
	F10-P1[d]	c.116T>C; c.1560_1561del	p.F39S; R521Qfs*44	F / -	18 y†	++++	-	-	-	+	+	+	profound
	F10-P2[e]	c.116T>C; c.1560_1561del	p.F39S; R521Qfs*44	F / -	6 y†	++++	-	-	-	+	+	+	profound

[a] Tepe et al., 2023; Family8, Subject10 (follow-up data)
[b] Tepe et al., 2023; Family8, Subject11 (follow-up data)
[c] Tepe et al., 2023; Family7, Subject9 (follow-up data)
[d] Tepe et al., 2023; Family 1, Subject 1
[e] Tepe et al., 2023; Family 1, Subject 2
†Deceased

Severity of GDD/ID: +, mild; ++, moderate; +++, severe; ++++, profound
Ambulation: +, ambulation with support; ++, independent ambulation

Abn.: Abnormal
Age f/u: Age at follow up
Cons.: Consanguinity
d: days
F: female
GDD: Global developmental delay
ID: Intellectual disability
NA: not available/not applicable
M: male
mo: months
y: years

B

**Gene**	**Variant**	**Classification**
INTS11	c.34G>A	Pathogenic [PP2, PP3, PP4, PP5, PM2, PS3]
	c.1219C>T	Pathogenic [PP2, PP3, PP4, PP5, PM2, PS3]
	c.649C>T	Pathogenic [PM2, PP2, PP3, PP4, PP5, PS3]
	c.1733A>G	Pathogenic [PM2, PP2, PP4, PP5, PS3]
	c.106C>T	Pathogenic [PM2, PM3, PP2, PP3, PP4, PS3]
	c.1295-9G>A	Pathogenic [PM2, PP4, PS3, PVS1]
	c.28+1406C>T	Likely pathogenic [PM2, PP4, PS3]
	c.1313C>T	Likely pathogenic [PM2, PP2, PP3, PP4, PS3]
	c.50G>T	Pathogenic [PM2, PP2, PP3, PP4, PP5, PS3]
	c.1041+3G>C	Likely pathogenic [PM2, PP4, PS3]
	c.656G>A	Likely pathogenic [PM2, PM3, PP2, PP4, PP5]
	c.936dup	Pathogenic [PM2, PP4, PP5, PVS1]
	c.568G>C	Likely pathogenic [PM2, PP2, PP3, PP4, PS3]
	c.1464+3G>C	Likely pathogenic [PM2, PP4, PP5, PS3]
	c.116T>C	Pathogenic [PM2, PM3, PP2, PP4, PP5, PS3]
	c.1560_1561del	Pathogenic [PM2, PM3, PP4, PP5, PS3, PVS1]

Very Strong

PVS1: Null variant in a gene where loss of function is a known disease mechanism.

Strong

PS3: Functional studies support a damaging effect.

Moderate

PM2: Variant is absent or extremely rare in large population databases (like gnomAD).

PM3: Detected in trans with another pathogenic variant in recessive disorders.

PM4: Protein length changes due to in-frame insertions/deletions in a non-repeat region.

Supporting

PP2: Missense variant in a gene with low benign variation and where missense variants are a common mechanism of disease.

PP3: Multiple lines of computational evidence (e.g., SIFT, PolyPhen) suggest a deleterious effect.

PP4: Patient's phenotype or family history is highly specific for a disease with a single genetic cause.

PP5: Variant reported as pathogenic by a reputable source, though without accessible evidence.

Fig. S1

Figure S1.
Clinical overview and variants of *INTS11*-mutated patients.
(**A**) Summary of patient characteristics (sex and age) and clinical parameters used to categorize individuals into phenotypic groups based on disease severity. (**B**) Variant classifications based on the American College of Medical Genetics and Genomics (ACMG) guidelines - *Pathogenic*, *Likely Pathogenic*, or *Variant of Uncertain Significance*, reflecting the likelihood that a given genetic variant contributes to disease (Richards *et al.*, 2015). Each classification is supported by specific ACMG criteria (e.g., PM2, PS3, PP4), representing different lines of evidence such as population frequency, computational predictions, and functional studies, including those performed in this study. Very Strong (PVS1), strong (PS3), moderate (PM2, PM3, PM4), supporting (PP2, PP3, PP4, PP5).

A

Gene	Participant ID	c-DNA	Protein	Sex/Cons.	Age f/u	GDD/ID	Ambulation	Spasticity	Ataxia	Seizures	Microcephaly	Abn. MRI	Phenotype
BRAT1	F11-P1[a]	c.185T>A	p.V62E	M / -	24 y	+	++	-	+	-	-	+	mild
	F11-P2[b]	c.185T>A	p.V62E	M / -	7 y	+	++	-	+	-	-	+	mild
	F12-P1[c]	c.1952G>A	p.R651H	M / +	5 y 2 mo	+	++	-	+	-	-	+	mild
	F12-P2[d]	c.1952G>A	p.R651H	F / +	4 y	+	+	-	+	-	-	+	mild
	F13-P1	c.2144T>A	p.V715E	F / -	4 y 8 mo	+	++	+	+	-	-	+	mild
	F14-P1[e]	c.2144T>A	p.V715E	F / +	5 y	+	+	-	+	-	-	+	mild
	F14-P2[f]	c.2144T>A	p.V715E	M / +	3 y	+	+	-	+	-	-	+	mild
	F15-P1	c.1930C>T; c.2324T>A	p.R644*; p.M775K	M / -	6 y	++	+	+	NA	+	-	+	moderate
	F16-P1	c.294dupA; c.491C>T	p.L99Tfs*92; p.A164V	M / -	4 y 1 mo	++	++	NA	+	-	-	+	moderate
	F17-P1	c.1172T>G	p.L391R	F / +	7 y 4 mo	+++	-	NA	+	+	+	+	severe
	F18-P1[g]	c.2125_2128del	p.F709Tfs*17	M / +	5 mo[†]	++++	-	+	NA	+	+	+	profound
	F19-P1[h]	c.1814A>C; c.2125_2128del	p.E605A; p.F709Tfs*17	M / -	2 y 8 mo	++++	-	-	-	+	+	+	profound

[a] Mahjoub et al, 2019; Sibling 1
[b] Mahjoub et al, 2019; Sibling 2
[c] Engel et al., 2023; Family 44, Patient 54
[d] Engel et al., 2023; Family 44, Patient 55
[e] Engel et al., 2023; Family 41, Patient 48
[f] Engel et al., 2023; Family 41, Patient 49
[g] Engel et al., 2023; Family 6, Patient 9
[h] *CASK* likely pathogenic missense variant (de novo); mosaic low level gain of chromosome 21 Karyotype
[†]Deceased

Severity of GDD/ID: +, mild; ++, moderate; +++, severe; ++++, profound
Ambulation: +, ambulation with support; ++, independent ambulation

Abn.: Abnormal
Age f/u: Age at follow up
Cons.: Consanguinity
d: days
F: female
GDD: Global developmental delay
ID: Intellectual disability
NA: not available/not applicable
M: male
mo: months
y: years

B

Gene	Variant	Classification
BRAT1	c.185T>A	Likely Pathogenic [PM2, PP4, PP5, PS3]
	c.1952G>A	Likely Pathogenic [PM2, PP4, PS3]
	c.2144T>A	Likely Pathogenic [PM2, PP3, PP4, PS3]
	c.1930C>T	Pathogenic [PM2, PP4, PP5, PS3, PVS1]
	c.2324T>A	Likely Pathogenic [PM2, PP4, PS3]
	c.294dupA	Pathogenic [PM2, PP4, PP5, PS3, PVS1]
	c.491C>T	Pathogenic [PM2, PM3, PP4, PP5, PS3]
	c.1172T>G	Likely Pathogenic [PM2, PP4, PS3]
	c.2125_2128del	Pathogenic [PM2, PP4, PP5, PS3, PVS1]
	c.1814A>C	Variant of Uncertain Significance [PM2, PP4]

Very Strong

PVS1: Null variant in a gene where loss of function is a known disease mechanism.

Strong

PS3: Functional studies support a damaging effect.

Moderate

PM2: Variant is absent or extremely rare in large population databases (like gnomAD).

PM3: Detected in trans with another pathogenic variant in recessive disorders.

PM4: Protein length changes due to in-frame insertions/deletions in a non-repeat region.

Supporting

PP2: Missense variant in a gene with low benign variation and where missense variants are a common mechanism of disease.

PP3: Multiple lines of computational evidence (e.g., SIFT, PolyPhen) suggest a deleterious effect.

PP4: Patient's phenotype or family history is highly specific for a disease with a single genetic cause.

PP5: Variant reported as pathogenic by a reputable source, though without accessible evidence.

Fig. S2

Figure S2.

Clinical overview and variants of *BRAT1*-mutated patients.

(**A**) Summary of patient characteristics (sex and age) and clinical parameters used to categorize individuals into phenotypic groups based on disease severity. (**B**) Variant classifications based on the American College of Medical Genetics and Genomics (ACMG) guidelines - *Pathogenic*, *Likely Pathogenic*, or *Variant of Uncertain Significance*, reflecting the likelihood that a given genetic variant contributes to disease (Richards *et al.*, 2015). Each classification is supported by specific ACMG criteria (e.g., PM2, PS3, PP4), representing different lines of evidence such as population frequency, computational predictions, and functional studies, including those performed in this study. Very Strong (PVS1), strong (PS3), moderate (PM2, PM3, PM4), supporting (PP2, PP3, PP4, PP5).

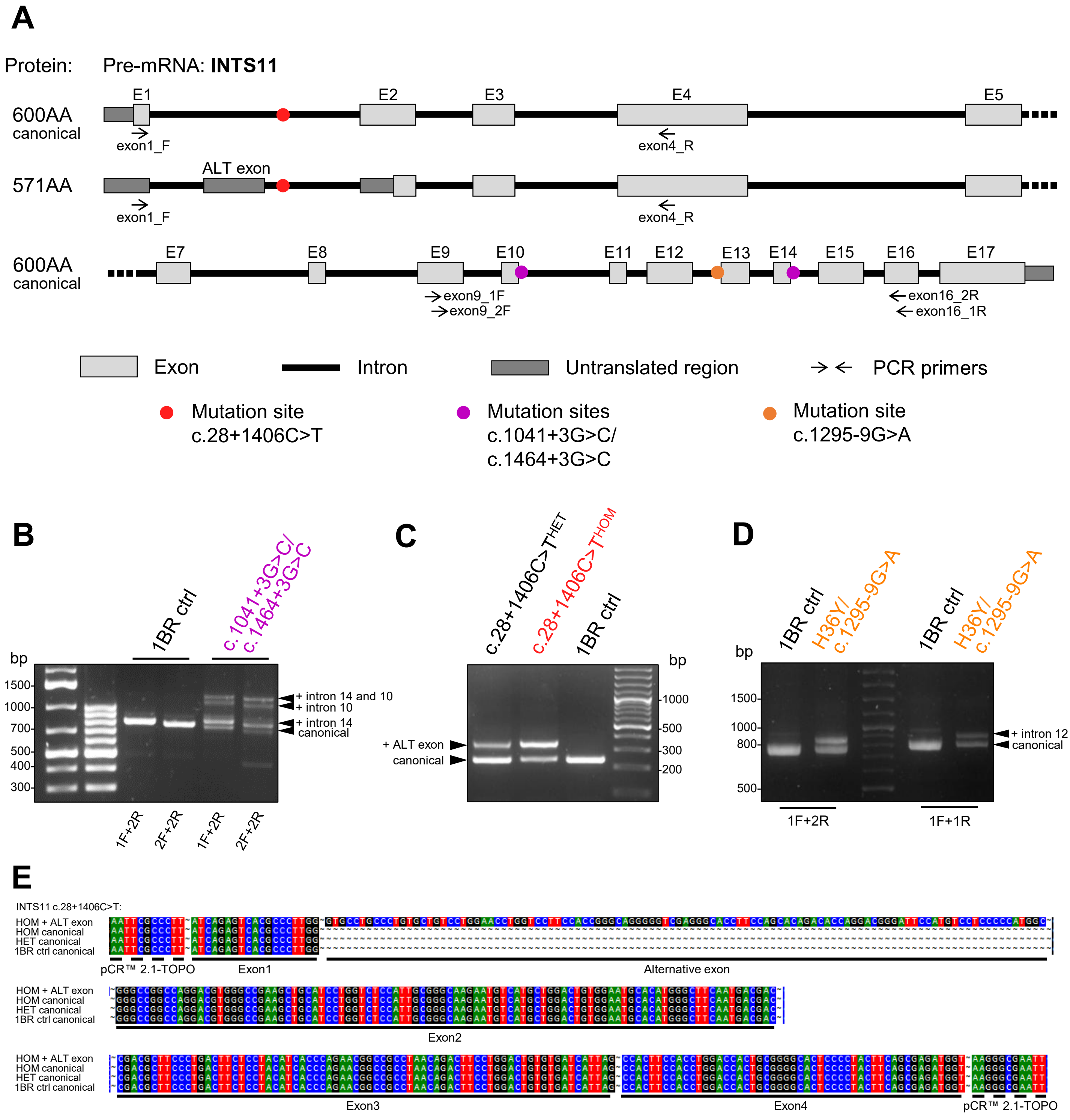
A
Protein:
Pre-mRNA: INTS11
600AA canonical
E1
E2
E3
E4
E5
exon1_F
exon4_R
571AA
ALT exon
exon1_F
exon4_R
600AA canonical
E7
E8
E9
E10
E11
E12
E13
E14
E15
E16
E17
exon9_1F
exon9_2F
exon16_2R
exon16_1R
Exon
Intron
Untranslated region
PCR primers
Mutation site c.28+1406C>T
Mutation sites c.1041+3G>C/ c.1464+3G>C
Mutation site c.1295-9G>A
B
1BR ctrl
c.1041+3G>C/ c.1464+3G>C
bp
1500
1000
700
500
400
300
+ intron 14 and 10
+ intron 10
+ intron 14
canonical
1F+2R
2F+2R
1F+2R
2F+2R
C
c.28+1406C>T^HET
c.28+1406C>T^HOM
1BR ctrl
bp
1000
500
300
200
+ ALT exon
canonical
D
1BR ctrl
H36Y/ c.1295-9G>A
1BR ctrl
H36Y/ c.1295-9G>A
bp
1500
1000
800
500
+ intron 12
canonical
1F+2R
1F+1R
E
INTS11 c.28+1406C>T:
HOM + ALT exon
HOM canonical
HET canonical
1BR ctrl canonical
pCR™ 2.1-TOPO
Exon1
Alternative exon
Exon2
Exon3
Exon4
pCR™ 2.1-TOPO

Fig. S3

Figure S3.

Affected splicing in patients with *INTS11* intronic mutations.

(**A**) Schematic representation of INTS11 pre-mRNA, showing the exons, introns, untranslated regions, and PCR primer binding sites. The c.28+1406C>T mutation site (*red*) is located in intron 1, the c.1041+3G>C and c.1464+3G>C mutation sites (*pink*) are in introns 10 and 14, respectively, and the c.1295-9G>A mutation site (*orange*) is in intron 12. (**B-D**) PCR analysis detecting alternative splicing in the three patients with mutations shown in (A); cDNA was used as the PCR template. (B) PCR detecting alternative splicing in the c.1041+3G>C/c.1464+3G>C patient, using unrelated 1BR sample as control. Patient mutations cause the retention of intron 10 and 14, or both. Two different primer pairs are used, as indicated: exon9_1F + exon16_2R, or exon9_2F + exon16_2R (*see Methods*). (C) PCR detecting alternative splicing in the c.28+1406C>T^{HOM} patient and the c.28+1406C>T^{HET} unaffected parent, with unrelated 1BR as control. The c.28+1406C>T mutation promotes the use of an alternative exon (canonical transcript: ENST00000435064.6; alternative transcript: ENST00000545578.5). (D) PCR detecting alternative splicing in the H36Y/c.1295-9G>A patient, with unrelated 1BR as control. The c.1295-9G>A mutation promotes splicing changes. Two different primer pairs are used, as indicated: exon9_1F + exon16_1R or exon9_1F + exon16_2R (*see Methods*). (**E**) Sanger sequencing of TOPO cloned PCR products shown in (C) confirms the use of the alternative exon in the presence of the c.28+1406C>T mutation. This alternatively spliced variant results in transcription starting from exon 3, as shown in (A), producing a 571 amino acids-long protein.

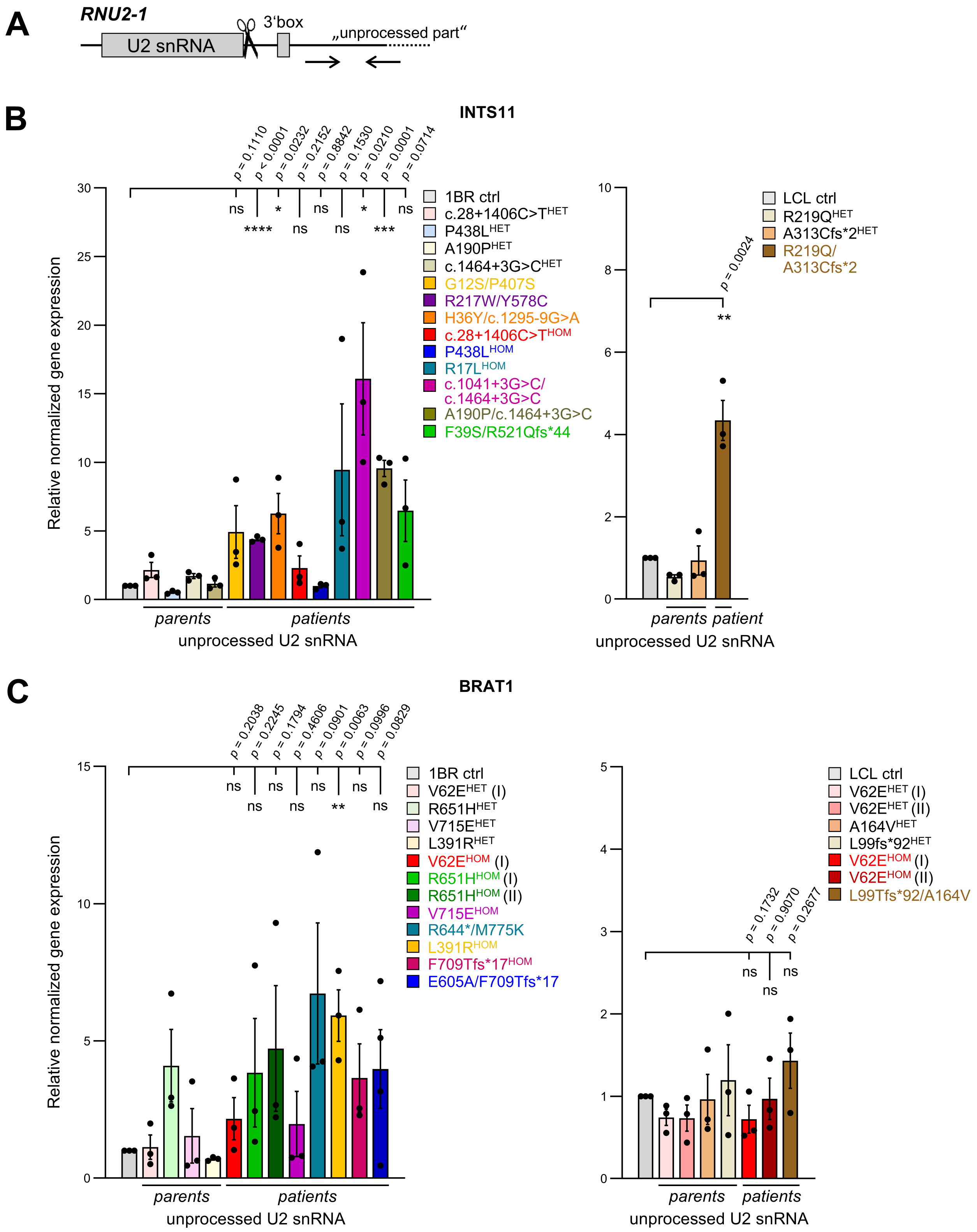

A
RNU2-1
U2 snRNA
3‘box
„unprocessed part“
B
INTS11
Relative normalized gene expression
p = 0.1110
p < 0.0001
p = 0.0232
p = 0.2152
p = 0.8842
p = 0.1530
p = 0.0210
p = 0.0001
p = 0.0714
ns
****
*
ns
ns
ns
*
***
ns
1BR ctrl
c.28+1406C>THET
P438LHET
A190PHET
c.1464+3G>CHET
G12S/P407S
R217W/Y578C
H36Y/c.1295-9G>A
c.28+1406C>THOM
P438LHOM
R17LHOM
c.1041+3G>C/
c.1464+3G>C
A190P/c.1464+3G>C
F39S/R521Qfs*44
parents
patients
unprocessed U2 snRNA
LCL ctrl
R219QHET
A313Cfs*2HET
R219Q/
A313Cfs*2
p = 0.0024
**
parents
patient
unprocessed U2 snRNA
C
BRAT1
Relative normalized gene expression
p = 0.2038
p = 0.2245
p = 0.1794
p = 0.4606
p = 0.0901
p = 0.0063
p = 0.0996
p = 0.0829
ns
ns
ns
ns
ns
ns
**
ns
1BR ctrl
V62EHET (I)
R651HHET
V715EHET
L391RHET
V62EHOM (I)
R651HHOM (I)
R651HHOM (II)
V715EHOM
R644*/M775K
L391RHOM
F709Tfs*17HOM
E605A/F709Tfs*17
parents
patients
unprocessed U2 snRNA
LCL ctrl
V62EHET (I)
V62EHET (II)
A164VHET
L99fs*92HET
V62EHOM (I)
V62EHOM (II)
L99Tfs*92/A164V
p = 0.1732
p = 0.9070
p = 0.2677
ns
ns
ns
parents
patients
unprocessed U2 snRNA

Fig. S4

Figure S4.

3'-end processing of U2 snRNAs in *INTS11*- and *BRAT1*-mutated patient cells.

(**A**) Schematic representation of RT-qPCR analysis design to detect unprocessed U2 snRNAs, encoded from *RNU2-1* gene. The diagram indicates the binding sites for primers used in RT-qPCR (*arrows*), the canonical Integrator-mediated cleavage site (*scissors*), and the downstream 3′box. (**B**) RT-qPCR analysis of unprocessed *RNU2-1* transcripts in control, parent and *INTS11*-mutated patient-derived fibroblasts (*left*) or LCLs (*right*). Data are shown as mean ± SEM ($N = 3$). Statistical significance was assessed using a two-sided Student's *t*-test (ns – not significant, *$p < 0.05$, **$p < 0.01$, ***$p < 0.001$, ****$p < 0.0001$). (**C**) RT-qPCR analysis of unprocessed *RNU2-1* transcripts in control, parent and *BRAT1*-mutated patient-derived fibroblasts (*left*) or LCLs (*right*). Data are shown as mean ± SEM ($N = 3$; $N = 4$ for E605A/F709Tfs*17 patient). Statistical significance was assessed using a two-sided Student's *t*-test (ns – not significant, **$p < 0.01$).

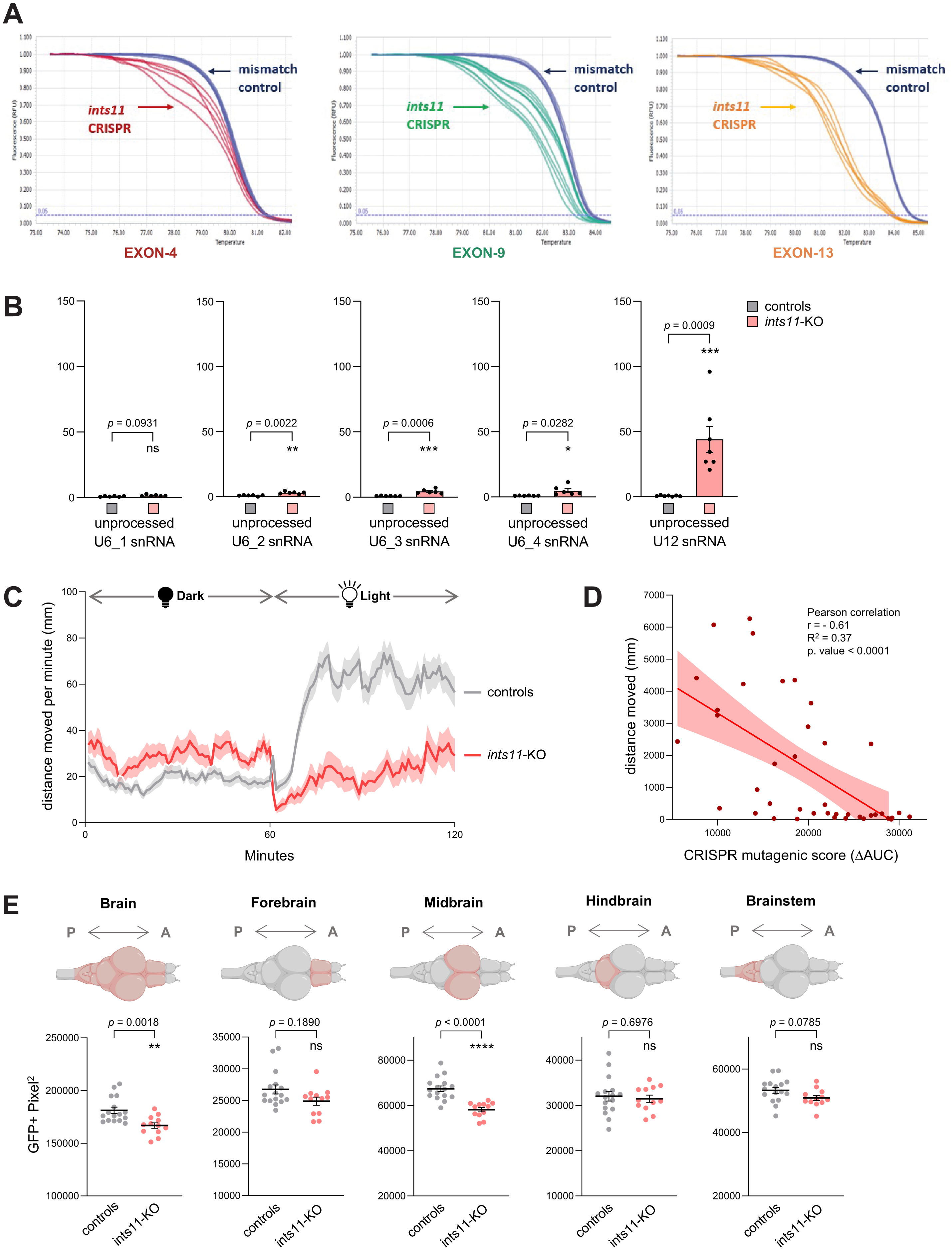
A
mismatch control
ints11 CRISPR
EXON-4
EXON-9
EXON-13
Temperature
Fluorescence (RFU)
B
controls
ints11-KO
p = 0.0931
ns
p = 0.0022
**
p = 0.0006
***
p = 0.0282
*
p = 0.0009
***
unprocessed U6_1 snRNA
unprocessed U6_2 snRNA
unprocessed U6_3 snRNA
unprocessed U6_4 snRNA
unprocessed U12 snRNA
C
Dark
Light
distance moved per minute (mm)
Minutes
controls
ints11-KO
D
Pearson correlation
r = - 0.61
R² = 0.37
p. value < 0.0001
distance moved (mm)
CRISPR mutagenic score (ΔAUC)
E
Brain
Forebrain
Midbrain
Hindbrain
Brainstem
P
A
GFP+ Pixel²
p = 0.0018
**
p = 0.1890
ns
p < 0.0001
****
p = 0.6976
ns
p = 0.0785
ns
controls
ints11-KO

Fig. S5

Figure S5.

Characterization of the *ints11*-deleted zebrafish model.

(**A**) All three *ints11* CRISPR guide RNAs (Exon 4, Exon 9, and Exon 13) exhibit a high-resolution melt (HRM) curve shift, confirming successful mutagenesis at each locus, while mismatch controls show no shift. (**B**) RT-qPCR analysis of 3' unprocessed snRNAs (*U6_1*, *U6_2*, *U6_3*, *U6_4*, *U12*) in 5 days post-fertilization (dpf) larvae. Data show fold-change relative to controls (ΔΔCt method). *P*-values from the Mann-Whitney test for U6_1 and U6_2, *p*-values from the two-sided Student's *t*-test for U6_3, U6_4 and U12: ns – not significant, $^{*}p < 0.05$, $^{**}p < 0.01$, $^{***}p < 0.001$ ($n > 35$, $N = 3$). (**C**) Distance moved (mm) binned per minute during a one-hour dark period and a one-hour light period. Sample sizes: controls ($n = 93$), *ints11*-KO ($n = 77$). (**D**) Pearson correlation graph showing the relationship between the mutagenic score of the *ints11*-CRISPant approach and the distance moved during the light phase. The mutagenic score was calculated as the differential area under the curve (ΔAUC) of high-resolution melting (HRM) profiles at the targeted locus, comparing controls and *ints11*-CRISPant larvae. (**E**) Quantification of different brain regions reveals a significant reduction in midbrain size, while the forebrain, hindbrain, and brainstem show no significant change in size. *P*-values from the Mann-Whitney test for whole brain and forebrain, *p*-values from the two-sided Student's *t*-test for midbrain, hindbrain and brainstem: ns – not significant, $^{**}p < 0.01$, $^{****}p < 0.0001$; sample sizes: controls ($n = 18$), *ints11*-KO ($n = 13$).

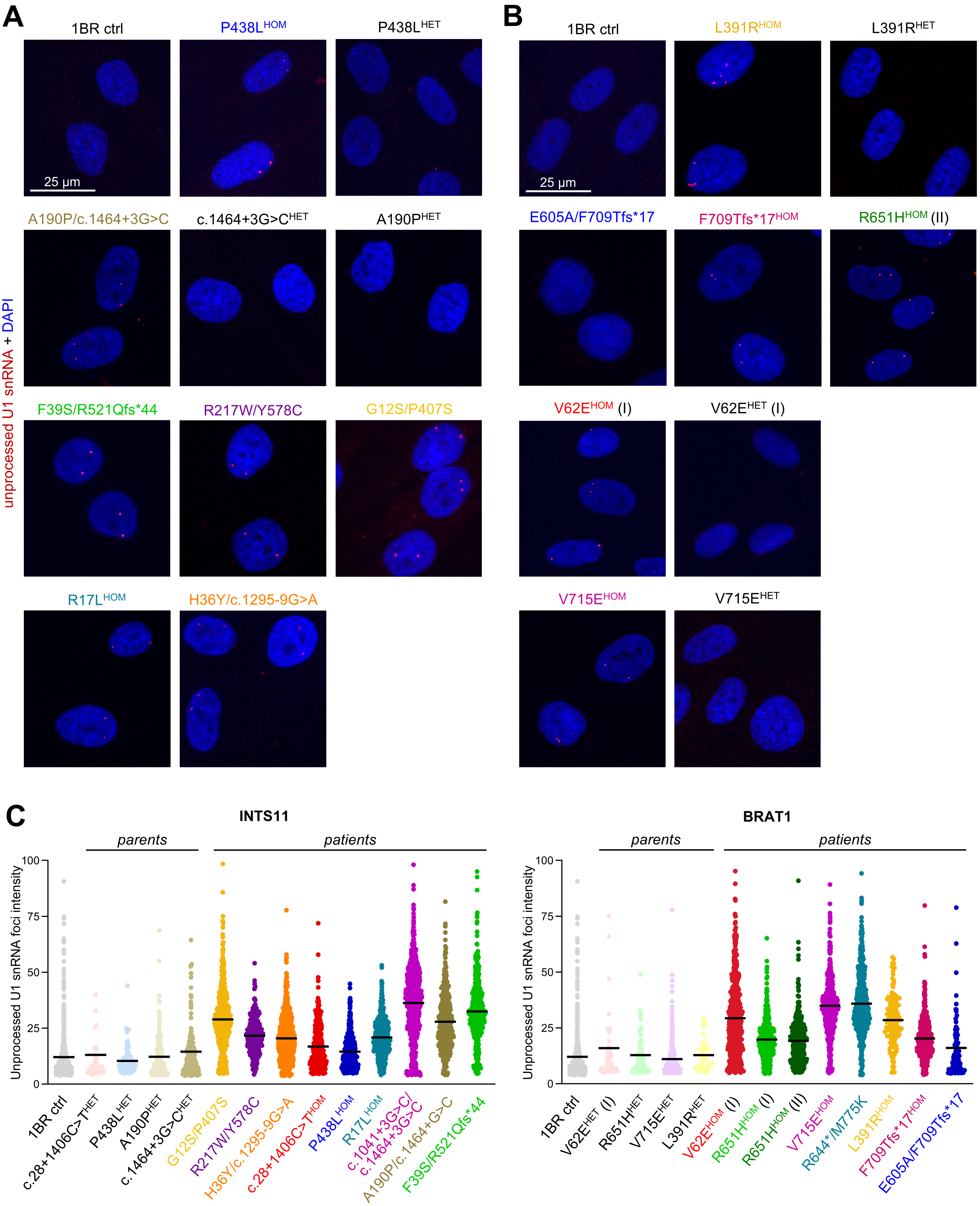
A
1BR ctrl
P438L HOM
P438L HET
25 µm
A190P/c.1464+3G>C
c.1464+3G>C HET
A190P HET
F39S/R521Qfs*44
R217W/Y578C
G12S/P407S
R17L HOM
H36Y/c.1295-9G>A
unprocessed U1 snRNA + DAPI
B
1BR ctrl
L391R HOM
L391R HET
25 µm
E605A/F709Tfs*17
F709Tfs*17 HOM
R651H HOM (II)
V62E HOM (I)
V62E HET (I)
V715E HOM
V715E HET
C
INTS11
parents
patients
Unprocessed U1 snRNA foci intensity
100
75
50
25
0
1BR ctrl
c.28+1406C>T HET
P438L HET
A190P HET
c.1464+3G>C HET
G12S/P407S
R217W/Y578C
H36Y/c.1295-9G>A
c.28+1406C>T HOM
P438L HOM
R17L HOM
c.1041+3G>C/ c.1464+3G>C
A190P/c.1464+G>C
F39S/R521Qfs*44
BRAT1
parents
patients
Unprocessed U1 snRNA foci intensity
100
75
50
25
0
1BR ctrl
V62E HET (I)
R651H HET
V715E HET
L391R HET
V62E HOM (I)
R651H HOM (I)
R651H HOM (II)
V715E HOM
R644*/M775K
L391R HOM
F709Tfs*17 HOM
E605A/F709Tfs*17

Fig. S6

Figure S6.

Nuclear accumulation of unprocessed U1 snRNAs in *BRAT1*- and *INTS11*-mutated patient-derived cells.

(**A-B**) Representative FISH images showing unprocessed U1 snRNAs (*red*) in *INTS11*-mutated (A) or *BRAT1*-mutated (B) fibroblasts, with the control and unaffected parent samples. Nuclei are stained with DAPI (*blue*). Scale bar: 25 μm. (**C**) Quantification of FISH signal intensity of unprocessed U1 snRNA foci in *INTS11*-mutated (*left*) and *BRAT1*-mutated (*right*) fibroblasts. Each dot represents one focus, measured across at least 121 nuclei ($n \geq 121$) per genotype in two independent experiments. Mean intensity is indicated.
